# Structural Proteomics Methods to Interrogate the Conformations and Dynamics of Intrinsically Disordered Proteins

**DOI:** 10.3389/fchem.2021.603639

**Published:** 2021-03-11

**Authors:** Rebecca Beveridge, Antonio N. Calabrese

**Affiliations:** ^1^ Department of Pure and Applied Chemistry, University of Strathclyde, Glasgow, United Kingdom; ^2^ Astbury Centre for Structural Molecular Biology, School of Molecular and Cellular Biology, Faculty of Biological Sciences, University of Leeds, Leeds, United Kingdom

**Keywords:** mass spectrometry, ion mobility, intrinsically disordered protein, hydrogen-deuterium exchange, crosslinking mass spectrometry

## Abstract

Intrinsically disordered proteins (IDPs) and regions of intrinsic disorder (IDRs) are abundant in proteomes and are essential for many biological processes. Thus, they are often implicated in disease mechanisms, including neurodegeneration and cancer. The flexible nature of IDPs and IDRs provides many advantages, including (but not limited to) overcoming steric restrictions in binding, facilitating posttranslational modifications, and achieving high binding specificity with low affinity. IDPs adopt a heterogeneous structural ensemble, in contrast to typical folded proteins, making it challenging to interrogate their structure using conventional tools. Structural mass spectrometry (MS) methods are playing an increasingly important role in characterizing the structure and function of IDPs and IDRs, enabled by advances in the design of instrumentation and the development of new workflows, including in native MS, ion mobility MS, top-down MS, hydrogen-deuterium exchange MS, crosslinking MS, and covalent labeling. Here, we describe the advantages of these methods that make them ideal to study IDPs and highlight recent applications where these tools have underpinned new insights into IDP structure and function that would be difficult to elucidate using other methods.

## Introduction

Interrogating the structure–function relationship of proteins and protein complexes has long been a productive area of scientific research, contributing to our understanding of biological processes and disease mechanisms and hence to the development of therapies. It was previously accepted that each protein has a specific three-dimensional conformation, composed of an intricate arrangement of secondary structure elements such as *α*-helices and *β*-sheets that dictates its function. More recently, however, it has emerged that not all proteins have a single specific conformation in their native state, and instead, they interconvert between multiple transient conformations, ranging from compact to extended, unhindered by energetic constraints (Wright and Dyson 2015). Such proteins, termed intrinsically disordered proteins (IDPs), are highly prevalent in biology, with bioinformatics studies indicating that the eukaryotic proteome is ∼20% disordered, with 36% of eukaryotic proteins containing >20% disorder and 12% carrying >50% disorder (Oldfield et al., 2005; Peng et al., 2015).

All proteins are dynamic to some extent, lying on a continuum between being mainly structured with minimum dynamics and mainly disordered with minimum structure. In proteins that are considered as “structured”, minimal dynamic behavior is an important feature that facilitates functions such as catalysis and macromolecular associations (Boehr et al., 2009), while spatial flexibility between structured domains can be afforded by disordered regions, such as those found in antibodies (Thielges et al., 2008). Disordered regulatory domains make excellent switches as they can be readily altered by posttranslational modifications, such as phosphorylation, for example, during different stages of the cell cycle or upon cell stress (Suryadinata et al., 2010). IDPs or proteins that contain intrinsically disordered regions (IDRs) are also players in liquid-liquid phase separation mechanisms, scaffolding the formation of intracellular biomolecular condensates that play key roles in cellular homeostasis, stress, and disease (Banani et al., 2017). IDPs exhibit binding plasticity, which means that they are often involved in cell signaling networks as they can bind to many protein partners transiently, but with high specificity (Wright and Dyson 2015). There are several ways in which IDPs can interact with other proteins. They may fold upon binding to their structured partners, also known as a “disorder-to-order” transition (Wright and Dyson 2009) or form a so-called “fuzzy complex,” in which the IDP samples various conformations on the surface of its binding partner (Sharma et al., 2015). A further advantage of disorder is that it economizes genome and protein resources, as the interface of an IDP in a protein–protein complex is similar to that formed between ordered proteins but consists of fewer residues from the disordered partner (Liu and Huang 2014). Overall, the flexible nature of IDPs and IDRs enables them to perform important functions in the cell that are complementary to the roles of ordered proteins.

IDPs often play an important role in signaling networks, thereby controlling cellular behavior, and their high propensity to aggregate means that they are often implicated in diseases such as cancer and neurodegenerative diseases (Uversky et al., 2008). For example, mutations in the protein p53, which binds other proteins mainly via disordered interactions, are found in many types of cancer including cancers of the colon, lung, breast, and brain (Hollstein et al., 1991), while mutations in the IDP breast cancer type 1 susceptibility protein (BRCA1) are, as the name suggests, often implicated in breast cancer (Semmler et al., 2019). Protein aggregation in the brain is often associated with neurodegenerative diseases, with *α*-synuclein (aSyn) aggregation being a pathogenic hallmark of Parkinson’s disease (Baba et al., 1998), and amyloid-beta (Aβ) and tau playing a role in the development of Alzheimer’s disease (Irvine et al., 2008).

Despite their biological and medical significance, a lack of suitable methods to interrogate IDPs means that they have remained underrepresented in the scientific literature. Resistance of IDPs and IDRs to crystallization renders them unsuitable for analysis by X-ray crystallography (XRC). Cryoelectron microscopy (cryo-EM) can effectively elucidate structures of mobile protein domains, although it remains challenging to elucidate copopulated states that differ with respect to the orientation of these domains, and IDRs are still missing from the structures (Murata and Wolf 2018). NMR is the most often used method to study IDPs as it can provide a signature for intrinsic disorder (low ^1^H and ^15^N amide chemical shift dispersion due to high solvent exposure) and provide information on transient secondary structure elements (usually alpha-helices) adopted in the overall conformational ensemble (Jensen et al., 2013). However, NMR cannot distinguish between multiple coexisting conformations, especially because rapid interconversions (on the NMR timescale) result in signal averaging. Other methods that are often used to study IDPs include small angle X-ray scattering (SAXS) (Kikhney and Svergun 2015), single molecule Förster resonance energy transfer (smFRET) (Kikhney and Svergun 2015), and computational methods such as Monte Carlo (MC) and molecular dynamics (MD) simulations (Baker and Best 2014; Mittal et al., 2014).

**FIGURE 1:**
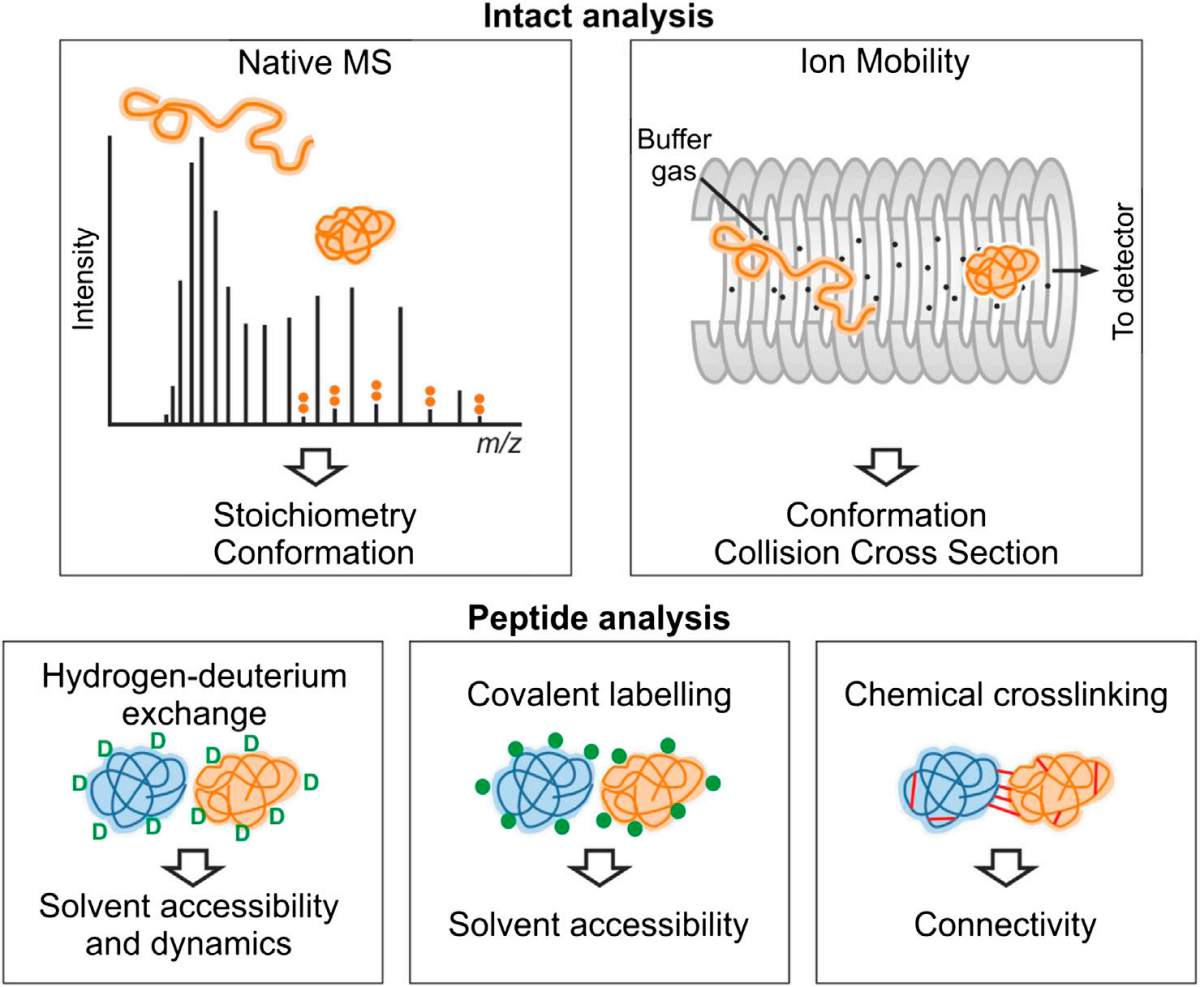
Overview of structural mass spectrometry methods. The information obtained from each experimental method is shown. Native MS and ion mobility analyses require the protein to be analyzed by MS intact under gentle ionization conditions that preserve noncovalent interactions. For an IDP, the CSD observed by native MS typically is multimodal consistent with multiple conformational states. These conformations (e.g., collapsed and extended) can be further separated by ion mobility. In hydrogen-deuterium exchange experiments, backbone amide hydrogen atoms undergo exchange with deuterium. Covalent-labeling methods involve modification of solvent accessible side-chains with a suitable probe, and in chemical crosslinking experiments, residue pairs in close spatial proximity are joined covalently. For these three methods, the protein(s) involved are typically proteolysed, and the labels are detected by analysis of the resultant peptide fragments.

Structural mass spectrometry (MS) methods (Figure 1), also known as structural proteomics methods, are undergoing an explosion in their application to study proteins and protein complexes, in part due to their wide applicability to many different protein types including structured, disordered, monomeric, and multimeric proteins (Politis and Schmidt 2018). Structural MS methods are able to capture transient interactions, can be used to study proteins and complexes up to the megadalton range, and can discern interactions in complex mixtures such as whole cell lysates and even intact cells (Liu et al., 2017). A key advantage of using MS-based methods to study IDPs is that they do not have any preference for the folded form of a protein that is displayed by XRC and cryo-EM, and they overcome size limitations afforded by NMR. They are often used in conjunction with other methods, providing complementary data that can be combined in order to predict structural preferences of proteins and complexes (Rout and Sali 2019).

Each method will be described more fully in the relevant sections, but in brief, native MS (nMS) reports on the binding stoichiometry of protein–protein and protein–ligand complexes on the basis of their mass (Beveridge et al., 2016). A key advantage of nMS over other techniques is that multiple binding stoichiometries can be simultaneously detected in complex mixtures, including those that are populated to a low extent, allowing detection of transient interactions (Leney and Heck 2017; Beveridge et al., 2020). nMS also provides information on the position of the protein on the structure-disorder continuum as the number of charges carried by a protein upon ionization is related to its fold (Beveridge et al., 2014). The shape of the charge state distribution (CSD) for an IDP can also reveal more detailed conformational preferences (Natalello et al., 2017; Santambrogio et al., 2019). nMS is often combined with ion mobility (IMMS) measurements or fragmentation methods for “top-down” mass spectrometry measurements (Stuchfield and Barran 2018; Donnelly et al., 2019). IMMS allows the size distribution of a protein or protein complex to be measured, in terms of its rotationally averaged collision cross section (CCS) (Sharon and Robinson 2007; Jurneczko and Barran 2011; Eyers et al., 2018). In top-down experiments, nonergodic fragmentation methods such as electron capture dissociation (ECD) or electron transfer dissociation (ETD) are implemented to break backbone peptide bonds, without disrupting any other structural elements. This can be used to locate binding sites of small molecules on proteins (Xie et al., 2006; Breuker and McLafferty 2008). With respect to IDPs, top-down MS has often been used to localize the binding of metal ions to a specific domain (Wongkongkathep et al., 2018). Conformational changes and binding events can also be localized using HDX-MS, which is a solution-phase technique that provides complementary information to nMS methods (Trabjerg et al., 2018). Differences in the uptake of deuterium are measured, which reflect changes in solvent accessibility of different regions of the protein upon ligand binding. Protein footprinting methods are additional solution phase approaches that label surface/solvent exposed residues to inform on protein structure and interactions. Crosslinking-MS (XL-MS) can be used to reveal sites of interactions, either within proteins or between proteins, informing on protein–protein interactions, and providing distance restraints to predict and validate protein models (Sinz et al., 2015; Leitner et al., 2016; Orbán-Németh et al., 2018).

As an update to the review published in 2013 (Beveridge et al., 2013), we will here provide an overview of the new advances in structural proteomics methods and their applications to the study of IDP behavior over the last 5 years.

## Native Mass Spectrometry

nMS is a widely applicable technique that informs on the stoichiometry and structural preferences of proteins and protein complexes. Upon “soft” transfer from solution into the gas phase by nanoelectrospray ionization (nESI), proteins can retain aspects of their conformation inside the mass spectrometer and the mass to charge (m/z) ratio of protein ions can be measured. The “mass” informs on the stoichiometry of a protein complex, and the number of charges that it carries informs on its structural preferences (Santambrogio et al., 2019). Structured proteins typically carry a low number of charges per unit mass and present over a narrow range of charge states, as their compact nature means that there is low solvent accessible surface area (SASA) upon which protons can be accommodated. In contrast, proteins that are unfolded have a larger SASA and can hence carry a higher number of charges (Kaltashov and Mohimen 2005; Testa et al., 2011; Beveridge et al., 2014). A protein that is completely denatured will have a Gaussian-like CSD with a high number of charges. IDPs, however, often exhibit residual structure which means that wide CSDs are typically observed, ranging from low to high charge states, which correspond to compact and extended conformations, respectively (Figure 1). Gaussian curves can be fitted to the deconvoluted CSDs of IDPs, which is postulated to inform on the number of conformational families adopted. In addition, the SASA can be estimated from the CSD of IDPs, which can be used as a constraint during modeling and IDP classification (Testa et al., 2011).

**FIGURE 2:**
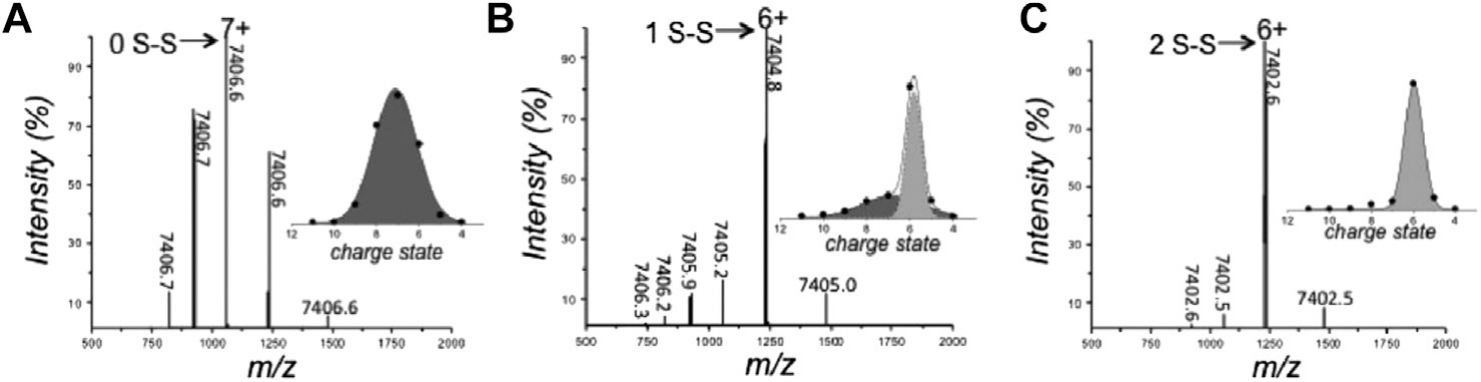
Analysis of the conformation and oxidative state of hCox17 species by native MS. nMS spectra of the hCox17 (10 μM) refolding reaction at pH 6.7. hCox17 species are labeled according to their charge state and number of disulfide bridges. Insets show the Gaussian fits to the observed CSDs. **(A)** Fully reduced hCox17 in the presence of 50 mM DTT. **(B)** Partially oxidized hCox17, 8 h after the removal of DTT. **(C)** Completely oxidized hCox17, 17 h after the removal of DTT. Figure adapted from (Fraga et al., 2017).

The rise in popularity of nMS is evidenced by the fact that it is now often used as part of a multimethod approach to study the biophysical characteristics of IDPs (Hagiwara et al., 2016; Fraga et al., 2017; Hamdi et al., 2017). Recently, Fraga et al. (2017) implemented nMS and other tools to interrogate the disorder-to-order transition of the human small copper chaperone, hCox17, that occurs upon the formation of disulfide bonds. Fully reduced hCox17 was desalted to remove DTT from the reaction mixture, and refolding was analyzed over a series of timepoints by combining information obtained from nMS with chromatography, fluorescence, circular dichroism (CD), Fourier transform infrared spectroscopy (FTIR), SAXS, and NMR. Of these methods, nMS affords the unique advantage of simultaneously determining the oxidation state of the protein and its conformational preferences. In its fully reduced form, hCox17 presents in a Gaussian-shaped CSD with charge states ranging from [M+5H]^5+^ to [M+9H]^9+^, with the highest intensity corresponding to [M+7H]^7+^ (Figure 2A). After 8 h of refolding, the protein is a mixture of fully reduced protein and a species which contains one disulfide bond. Fitting Gaussian curves to this deconvoluted spectrum reveals a compact conformation with high intensity centered around [M+6H]^6+^ (containing one disulfide bond) and an extended one at much lower intensity centered around [M+7H]^7+^ (reduced) (Figure 2B). This change in CSD suggests that a significant compaction occurs upon formation of the first disulfide bond. After 17 h refolding, the native form, which contains two disulfide bonds, accumulates, but the main charge state remains at [M+6H]^6+^ (Figure 2C), indicating that no significant further compaction occurs. In agreement with this assertion from nMS, SAXS experiments discerned that fully reduced Cox17 has a radius of gyration (R_g_) of 24.2 ± 0.3 Å which compacts to 21 Å that is stable for the following 4 h. After 6 h, there is a small decrease of 1 Å. Differences in the refolding times are likely a result of differential solution conditions employed (i.e., pH was 8.4 and 6.7 for SAXS and nMS, respectively). This study demonstrates the suitability of studying preferred protein conformations with nMS, the agreement of nMS with solution phase SAXS measurements, and the complementarity of nMS with other methods such as NMR and CD, which reveal secondary structure elements. A similar study was undertaken by Snijder et al. (2015), in which nMS and IMMS was used to delineate the conformational preferences of different oxidation states of human alpha-defensin.

Structural properties of the flexible dimer formed between the intrinsically disordered N^TAIL^ domain and the phosphoprotein X domain (P^XD^) from measles virus (MeV) were revealed by CSD analysis (D’Urzo et al., 2015). Each component was analyzed with nMS, which reported a wide CSD for each protein. Fitting of Gaussian curves to the deconvoluted spectra revealed a mixture of three conformational families for N^TAIL^ and two for P^XD^. Upon mixing the two proteins at equimolar concentrations, the dominant peaks in the spectrum corresponded to the unbound proteins, with lower-intensity peaks corresponding to a 1:1 dimer, reflecting the transient nature of the interaction. Interestingly, the dimer also exhibits a wide CSD ranging from [D+9H]^9+^ to [D+21H]^21+^, indicating that the significant disorder is retained upon complex formation. The CSD corresponds to approximately two conformations, an open and a closed, with estimated SASA values of 16.1 nm^2^ and 9.5 nm^2^, respectively. These values, along with the CCSs measured by IMMS, were used as distance restraints in computational modeling, resulting in the first structural model of this fuzzy complex.

CSD analysis formed an important part of the study into the conformational preferences of the disordered C-terminal domain of p27 (p27^C^) and two of its permutants (Beveridge et al., 2019a): one in which oppositely charged residues were more evenly spaced along the protein sequence (p27^C^-κ14) and the other in which oppositely charged residues are linearly segregated (p27^C^-κ56) (Das and Pappu 2013; Das et al., 2016). nMS provided a clearer distinction between the conformational preferences of the permutants than could be achieved with other biophysical techniques, revealing the more disordered nature of p27^C^-κ14 with respect to the wildtype and the compaction of p27^C^-κ56. While these systems were further interrogated by IMMS (see below), the CSDs alone were sufficient to inform on the conformational preferences of these disordered proteins and their response to differential ionic strengths of the solutions from which they were ionized (lower ionic strength had a negligible effect on p27^C^-κ14, promoted extended conformations of p27^C^-WT and caused further compaction of p27^C^-κ56).

CSD analysis also provided important information during the study of MAGE-A4 which is a key target for cancer therapy (Hagiwara et al., 2016). In this study, nMS was used alongside structure-based homology modeling, circular dichroism, and NMR to discern the conformational effects of nine mutations that have been implicated in cancer. nMS reported a reduction in charge state range of all mutants compared to the wildtype, centered around lower charge states, suggestive of a more compact form being dominant in solution. NMR analysis indicated that MAGE-A4 contains both structured and disordered regions and reported small alterations to its hydrophobic core in the mutants studied. As in the p27^C^ study described above, differences identified with nMS were more apparent than with any other methods. Understanding the behavior of MAGE-A4 at the molecular level is the first step required for its exploitation in translational research toward cancer therapies.

nMS is also useful in discerning the stoichiometry of protein complexes and can be particularly beneficial in the study of IDP-containing complexes, as their flexibility and transient binding nature often precludes analysis by alternative techniques. Recently, Stephani et al. (2020) used nMS to study the protein C53, which consists of two globular domains that are joined by a disordered linker region that contains ATG8 interacting motifs. The stoichiometry of the complex formed between ATG8 and C53 remained elusive, until nMS revealed that complexes comprising 1:1 and 1:2 ratios of C53 to ATG8 were both detected.

### Ion Mobility Mass Spectrometry

nMS is frequently coupled with ion mobility spectrometry, which separates protein conformations according to their size. Three types of ion mobility will be described in this section: drift tube ion mobility-MS (^DT^IMMS), traveling wave IMMS (TWIMMS), and trapped ion mobility mass spectrometry (TIMS).

#### Drift Tube Ion Mobility Mass Spectrometry

^DT^IMMS requires the simplest instrumentation of all IMMS types. Protein ions are pulsed into a drift tube, across which is applied a weak electric field that pulls the ions through the cell (Gabelica and Marklund 2018). The ions are hindered by collisions with an inert buffer gas such as helium, and since larger ions experience a higher number of collisions, extended protein conformations are slowed down to a greater extent than compact conformations. The arrival time distribution (ATD) of the ions exiting the cell is recorded, and when combined with m/z measurement, their sizes can be directly converted to a rotationally averaged collision cross-section (CCS) that reveals low-resolution information on the conformational distribution of an IDP (Stuchfield and Barran 2018).

**FIGURE 3:**
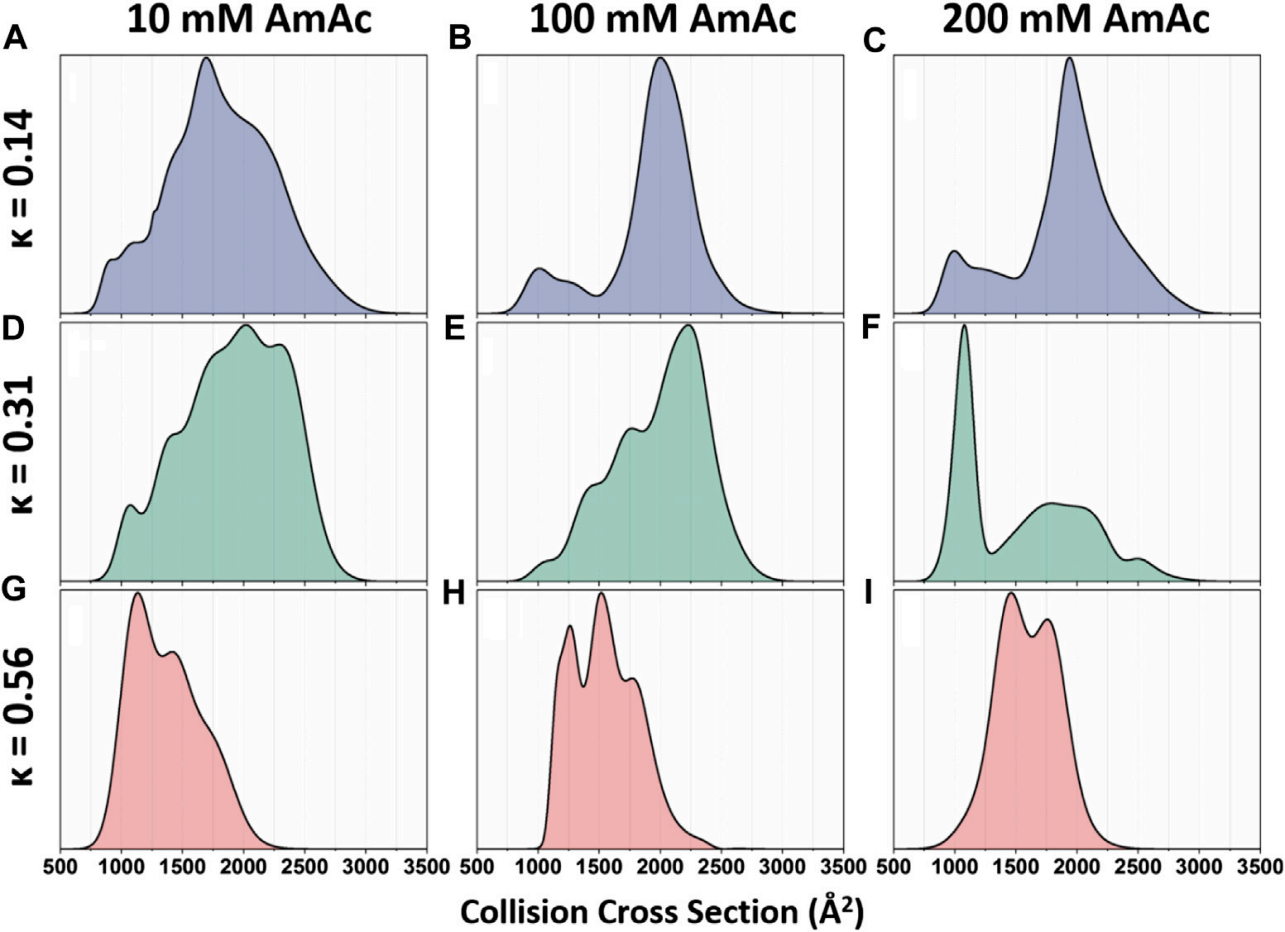
Global collision cross-section distributions of all three p27 permutants sprayed from 10 mM, 100 mM, or 200 mM ammonium acetate. Global collision cross-section distributions are shown for the **(A–C)** p27^C^-κ14 (blue), **(D–F)** p27^C^-WT (green), and **(G–I)** p27^C^-κ56 (red) permutants. Global CCSDs are obtained by combining CCSDs of individual charge states into single feature-rich distributions. Oppositely charged residues are well mixed in sequences with low κ values, whereas oppositely charged residues are segregated in sequences with high κ values (Das and Pappu 2013). Figure adapted from (Beveridge et al., 2019a).

^DT^IMMS was used to thoroughly interrogate the structural preferences of p27^C^-WT and the permutants p27^C^-κ14 and p27^C^-κ56, described above (Beveridge et al., 2019a). Analysis of CCS distributions allowed quantitation of conformational alterations in the charge pattern variants and their response to salt concentrations (Beveridge et al., 2019a). p27^C^-κ14 is highly dynamic when sprayed from a low salt solution (10 mM ammonium acetate), displaying a wide CCS distribution that has little preference for any particular shape (Figure 3A). Upon increased salt concentrations (100 mM and 200 mM ammonium acetate for middle and high salt concentration solutions, respectively), p27^C^-κ14 is stabilized in extended conformations with CCSs centered around 2000 Å^2^, with just a small proportion of molecules in compact conformations (750–1,500 Å^2^) (Figures 3B,C). p27^C^-WT is also highly dynamic when sprayed from the low salt solution (Figure 3D) and is stabilized in an extended conformation above 2000 Å^2^ in the middle salt solution (Figure 3E), with the smaller conformations below 1,500 Å^2^ representing a slightly higher relative abundance than for p27^C^-κ14. When sprayed from a high salt solution, a compact conformation centered around 1,000 Å^2^ becomes the most abundant for p27^C^-WT, with extended conformations above 1,500 Å^2^ in much lower signal intensity (Figure 3F). p27^C^-κ56 adopts compact conformations (highest intensity around 1,000 Å^2^) when sprayed from a low salt solution (Figure 3G). The middle salt solution causes an increase in size of the protein, with highest intensity increasing to 1600 Å^2^ and both the minima and maxima of the CCS distribution increasing by 250 Å^2^ (Figure 3H). The high salt concentration leads to even further expansion (Figure 3I). This work demonstrates the strength of ^DT^IMMS in discerning the behavior of IDPs and their response to differential solution conditions from which they are sprayed. In an additional work on p27, the interaction mechanisms of p27 constructs with the cellular binding partner Cdk2/cyclin A were interrogated. It was found that the KID domain of p27 (p27^KID^), which folds upon binding to Cdk2/cyclin A, causes the rigid dimer to become more dynamic. As expected, full-length p27 (p27^FL^) is tamed upon binding, with the most extended conformations no longer being accessible upon complex formation (Beveridge et al., 2019b). In this work, the strength of IMMS is its ability to visualize both the bound and unbound form of an IDP, without the preference for the bound form that is displayed by many techniques.

Bowers and coworkers have developed ^DT^IMMS methods to study the conformations and aggregation of Aβ, a protein fragment that is the main component of amyloid plaques found in Alzheimer’s disease patients (Murphy and LeVine 2010). In 2011, the conversion of Aβ-derived peptides from disordered monomers to structured beta-sheet amyloid fibrils was traced (Bleiholder et al., 2011), revealing differential assembly pathways for the different peptides. In the last five years, ^DT^IMMS has provided extensive characterization of Aβ including delineating mechanisms of aggregation inhibition (Zheng et al., 2016; Downey et al., 2019) and detecting prominent oligomers (Economou et al., 2016). Work by the same group provided the first experimental evidence of an interaction between two IDPs whose aggregation is associated with diseases in different tissues, namely, islet amyloid polypeptide (IAPP), implicated in type 2 diabetes, and a fragment of tau, implicated in Alzheimer’s (Arya et al., 2019). They also shed further light on the coaggregation of nonhomologous peptides derived from PrP and IAPP, determining that the PrP peptide promotes the transition of IAPP into its aggregation-prone *β*-hairpin conformation (Ilitchev et al., 2018).

#### Travelling Wave Ion Mobility Mass Spectrometry

Due to TWIMMS being implemented into the first commercially available integrated IMMS device (Synapt, Waters, United Kingdom), this has become the most widely used type of IMMS separation for protein structural studies (Illes-Toth et al., 2015; Konijnenberg et al., 2016; Van der Rest et al., 2017). The Synapt G1 was released in 2006, and more recent versions have provided users with enhanced resolution and sensitivity. During ion separation via TWIMMS, traveling waves propel ions through the ion mobility device at different speeds according to their charge and size (Lanucara et al., 2014). Unlike ^DT^IMMS, the direct calculation of CCS from TWIMMS data is not possible, and CCSs can only be calculated by careful calibration with known standards (Ruotolo et al., 2008; Bush et al., 2012). TWIMMS has provided important insights into the metal-induced conformational changes of human metallothionein-2A (Chen et al., 2014; Dong et al., 2018) and into the conformational variability of the NAC complex which helped to describe its nature of substrate recognition during chaperone activity (Martin et al., 2018). Additionally, TWIMMS effectively demonstrated that the disordered tails of histone proteins play an important role in stabilizing the core regions of the functionally important histone dimers (Saikusa et al., 2015).

TWIMMS was recently used to discern how the small EDRK-rich factor 1 (SERF) protein increases the fibrillation rate of Aβ_1–40_ and aSyn. SERF and its homologues have been shown to accelerate amyloid formation of many amyloidogenic proteins in vitro, but the mechanism behind this had remained elusive. SERF was analyzed alone and in complex with Aβ_1–40_ or aSyn by IMMS to investigate protein–protein interactions that could affect the critical early steps of amyloid formation, leading to altered aggregation rates (Meinen et al., 2019). Monomeric SERF displays a wide CSD (charge states [M+5H]^5+^ to [M+15H]^15+^) and a wide CCS distribution (900–2,200 Å^2^), reflective of its highly disordered nature. In a 1:1 mixture of SERF with Aβ_1–40_, complexes were identified with 1:1, 1:2, and 2:1 stoichiometries, while in an equimolar mixture with aSyn, 1:1, 1:2, 2:1, and 2:2 complexes were detected. IMMS reported that all these complexes exhibit extreme heterogeneity, with much wider CCS distributions than expected from complexes of similar sizes. For example, the 2:2 SERF:aSyn complexes have conformations that are as large as antibodies (up to 7,000 Å^2^), despite being approximately one-third of the mass. Overall, this IMMS data provided the first evidence that SERF forms dynamic complexes with Aβ_1–40_ and aSyn, in which the authors used to propose a model in which SERF provides a binding surface that increases the pool of conformations that these proteins can explore to accelerate primary nucleation. It has been tentatively postulated that accelerating amyloid formation with SERF may reduce the time that the proteins spend as neurotoxic intermediates.

An exciting recent development is the commercialization of cyclic IMMS instrumentation (Waters, United Kingdom), in which the ions are separated by passing them around a circular ion mobility cell. The number of passes around the cell can be controlled by the user to increase the effective distance over which the ions are separated, hence, increasing the resolution of the IM measurement, before the ions are ejected into the mass analyzer (Eldrid et al., 2019; Giles et al., 2019; Ujma et al., 2019). This was used by Thalassinos and coworkers (Eldrid et al., 2020) to compare the gas phase behavior of a structured protein (cytochrome c) and an IDP (human islet amyloid polypeptide hIAPP, also referred to as amylin), discovering differences in the gas-phase unfolding of the distinct protein types.

#### Trapped Ion Mobility Spectrometry

During TIMS separation, ions are propelled through the IMS cell with a gas flow and hindered by an electronic gradient in the opposite direction. This means that increased charge slows the ions down, and larger surface area speeds them up, in contrast to how separation is achieved in ^DT^IMMS (Ridgeway et al., 2018). Garabedian et al. (2018) used TIMS to study the conformational preferences of the intrinsically disordered AT-HOOK peptide, which is the DNA binding region of the mammalian high mobility group protein (HMGA2). This disordered protein influences the remodeling of chromatin structure, thereby regulating the expression of certain genes, and is a target in several cancer therapies. The structural effects of seven separate point mutations and their influence on complex formation of the peptide with AT-rich regions of DNA hairpins were elucidated. Specifically, the RGRP core (residues 4–7) was found to be essential for stabilizing peptide-DNA interactions, with the weakest complex being formed with the R6A mutant, suggesting that this basic residue is important in binding DNA.

#### Ion Mobility Mass Spectrometry Studies of Tau

A protein that has come under particular scrutiny by IMMS in recent years is tau (Eschmann et al., 2015; Ganguly et al., 2015; Jebarupa et al., 2018; Ahmadi et al., 2019), a 441 residue protein comprising disordered N-terminal (∼200 residues) and C-terminal (∼80 residues) regions (Cleveland et al., 1977). The central region of the protein is thought to have residual structure and form the core of amyloid fibrils (Fitzpatrick et al., 2017). Deposition of tau aggregates in the neurons and glia of the brain is a hallmark of neurodegenerative disease (Ferrer et al., 2014). Eschmann et al. (2015) used ^DT^IMMS in combination with transmission electron microscopy (TEM), thioflavin T (ThT) fluorescence, electron paramagnetic resonance (EPR), and Overhauser dynamic nuclear polarization (ODNP) to study a peptide fragment of tau called R2 (residues 273–284) that contains a nucleating six-residue segment and forms cross beta-sheet fibrils, similar to those found in pathological aggregation. They discovered differences in the relative abundances of monomer, dimer, and conformational populations that dictate the propensity for fibril formation. In a separate study, Ganguly et al. (2015) compared this same peptide with a second peptide that contains a similar nucleating six-residue segment (R3 306–317), as well as a mutant of the first peptide (R2 delta-K280) that is associated with a neurodegenerative tauopathy. IMMS determined that R3 homodimers are the most stable, followed by heterodimers containing R3, and the least stable dimers are homodimers of R2wt and R2-dK280. ThT assays showed that R3 has a higher propensity to aggregate than wtR2 or R2-dK280. R2-dK280 binds more strongly to R3 than R2wt does, suggesting a possible mechanism for the tauopathy. In a study by Jebarupa et al. (2018), the largest human tau isoform was analyzed with TWIMMS, which uncovered the extreme plasticity of the protein, with conformations ranging from compact to extended. Compact conformations were removed upon acidification of the starting solution, whereas alkaline pH caused compaction due to charge neutralization. Changes in structure at both high and low pH can be linked to the higher propensity to aggregate under both conditions. The interaction of tau with a molecular tweezer assembly modulator has been investigated by TWIMMS combined with top-down MS (Nshanian et al., 2019).

### Native Top-Down Mass Spectrometry

As described above, protein–ligand interactions can be maintained in the gas phase upon careful ionization from their starting solution, allowing determination of binding stoichiometry. Moreover, during top-down experiments, electron capture dissociation (ECD) or electron transfer dissociation (ETD) is used to cleave covalent backbone bonds of proteins without disturbing noncovalent interactions, including those between protein and ligand. This has allowed site localization of ligand and metal binding to proteins (Xie et al., 2006). Early work investigating the potential of top-down MS to localize protein–ligand interactions focused on the complex formed between aSyn and spermine (Xie et al., 2006). Upon fragmentation of aSyn via ECD, product ions were identified that retained the bound spermine, allowing localization of the spermine binding site to residues 106–138, consistent with NMR studies. More recently, top-down MS was used to characterize cobalt and manganese binding to aSyn (Wongkongkathep et al., 2018), as these metals have been implicated in accelerated aSyn aggregation. nMS revealed that at a 1:5 protein to metal concentration, aSyn is present in the unbound form and with one or two bound metal ions, with the same binding patterns observed for both manganese and cobalt. Upon ECD fragmentation of the 1:1 aSyn-cobalt species, the majority of the C-terminal fragments have cobalt bound, while the majority of N-terminal fragments up to residue 118 do not, suggesting that the cobalt binding site is located in the C-terminal tail. The binding of manganese occurred via similar interactions. Interestingly, due to the binding of cobalt and manganese to aSyn being governed by electrostatic interactions, their complexes were highly stable in the gas phase and even survived high energy dissociation via collisional activation dissociation (CAD). CAD revealed the same site localization as ECD, but differences in the fragmentation of the cobalt-aSyn complex with respect to the apo form suggested a structural rearrangement upon cobalt binding, a hypothesis that was confirmed with IMMS experiments. To test the assertions in site localization made by ECD analysis, three truncated aSyn variants (1–60, 61–140, and 96–140) were also characterized. As expected, cobalt and manganese bind to both the 61–140 and 96–140 fragments, but not to the N-terminal 1–60 fragments.

Top-down MS was also used to interrogate the binding of the “molecular tweezer” CLR01 to tau (Nshanian et al., 2019). CLR01 has been found to inhibit aggregation of amyloidogenic proteins without toxic side effects (Sinha et al., 2011; Acharya et al., 2014; Vöpel et al., 2017). It binds specifically to lysine residues in disordered proteins and remodels their assembly into nontoxic, nonamyloidogenic structures. nMS shows that CLR01 binds to tau in a 1:1 stoichiometry. A 4R-repeat domain fragment (residues 257–239, 11 kDa) also binds with 1:1 stoichiometry. Top-down ECD-MS of the 10+ charge state of the CLR01-bound 4R-fragment revealed c- and z-product ions that imply that the core of the tau fragment is the likely region of ligand binding, between K294 and K331, that includes lysine residues at positions 294, 298, 311, 317, 321, and 331. This corresponds to the microtubule-binding domain, which spans the two nucleating peptides, as described above, which are essential for seeding. Sequence coverage drops from 61% to 46% for the 4R-repeat domain fragment, suggesting compaction of the protein, which is consistent with IMMS data showing compaction of the 9+, 8+, and 7+ charge states. Interestingly, IMMS studies also indicate that CLR01 causes compaction of Aβ-dimers and tetramers (Zheng et al., 2015).

## Hydrogen-Deuterium Exchange-Mass Spectrometry

HDX-MS is a sensitive and versatile method that locates changes in solvent accessibility of proteins and protein complexes, thereby reporting on structural differences and alterations in flexibility (Trabjerg et al., 2018). In an HDX-MS experiment, the protein of interest is diluted into a deuterated buffer, and the deuterium from the solvent is allowed to exchange with the backbone amide hydrogens for a given amount of time. The reaction is quenched by the addition of a low-pH buffer, and the protein is then digested with an acid-stable protease (usually pepsin). Finally, the deuterium uptake of each peptide is measured via liquid chromatography- (LC-) MS. This allows identification of regions of protein sequence that are more or less exposed upon structural perturbation, for example, upon ligand/cofactor binding (Beveridge et al., 2016). Given that the analysis is performed on the digested peptides, there are no limitations on the maximum size of the system that can be studied, and HDX-MS can be applied to study IDRs and IDPs.

In recent years, huge improvements have been made to HDX methodologies that have increased its applicability to study residual structure in IDPs. Efforts toward this have included reducing the temperature and pH of the experiments to slow exchange (Goswami et al., 2013) and specialist apparatus to allow fast mixing on the msec timescale. Fast mixing apparatus includes microfluidic chip-based technologies (Liuni et al., 2010; Yamamoto et al., 2011; Rob et al., 2012; Svejdal et al., 2019) and quench-flow systems (Rist et al., 2005; Keppel et al., 2011; Keppel and Weis 2013; Walters et al., 2013). One such quench-flow apparatus was designed and constructed by Keppel and Weis using solely off-the-shelf components and allows HDX measurements on the 50–5000 msec timescale (Keppel and Weis 2013). Briefly, three syringes are used to deliver the sample (in H_2_O buffer, syringe 1), label (D_2_O buffer, syringe 2), and quench (syringe 3). Deuteration is initiated in the first mixing “T” where the sample in H_2_O buffer is combined with D_2_O buffer. The two flows are mixed at an angle of 180° and the resulting reaction mixture exits the tee through a short delay line before it is combined with quench at the second “T” mixer. A key advantage of this approach is that the exchange time can be simply adjusted by altering the flow rate. The quenched sample can be immediately frozen in liquid nitrogen or the system can be connected directly to an LC, ESI-MS, or a pepsin column for online sample labeling and analysis. The authors tested this msec HDX setup by comparing the deuterium uptakes of the CREB binding protein CBP^2059–2117^ alone and in complex with the activator of retinoid and thyroid receptor, ACTR^1018–1088^. Unbound CBP is known to be a molten globule that populates two folded states, but in a previous work using a conventional HDX approach, unstructured regions of CBP could not be distinguished from the structured core since exchange was almost complete along the full length of the protein within 10 s (Keppel et al., 2011). Shifting to an msec exchange timescale provided a distinction between the N- and C-terminal domains, where the exchange is at the near-intrinsic rate, and the helical core of the protein, where the exchange is significantly slower. Upon binding ACTR, HDX of many peptides in the core region of CBP became too slow for detection in the 3500 ms time course. The C- and N-terminal domains also incurred increased protection, suggesting that the fraying ends of helices one and three were stabilized upon binding of ACTR. Such details could not be uncovered with conventional HDX; hence, shifting the experiment to an msec timescale is highly advantageous in the analysis of IDPs. In subsequent research, Keppel and Weis used the quench-flow apparatus to study a similar construct of ACTR (residues 1,023–1,093) to probe its residual helicity (Keppel and Weis 2015). They quantified the rate of HDX in terms of midpoint values for each peptide, which represent the time required to reach 50% deuteration, relative to a fully deuterated control (*t*_50%_) (Al-Naqshabandi and Weis 2017). The fastest exchanging peptide is from the N-terminus of the protein, which is 50% deuterated at 0.2 s and is almost fully exchanged by 3.5 s. In contrast, a peptide corresponding to a portion of the protein that has been shown to form a residual helix by NMR takes 1.6 s to reach 50% and is only 65% deuterated after 3.5 s. After residue-averaging the data from overlapping peptides, there is a good correlation between residues with *t*_50%_ values greater than 0.5 s and the regions of ACTR that become helical in complex with CBP (Demarest et al., 2002). There is also a good agreement between the regions that display protection in HDX and the regions of transient helices that have been measured by NMR of the protein in isolation (Kjaergaard et al., 2011), but with lower spatial resolution. Nevertheless, HDX has the advantage of being applicable to nonisotopically labeled protein and to much larger proteins than NMR. Additional applications of HDX by the Weis group include investigating the influence of crowding on the structure of IDPs (Rusinga and Weis 2017a; Rusinga and Weis 2017b), which requires extensive clean-up of the protein to remove artificial crowders prior to MS.

Kish et al. developed a fully automated online flow mixing and quenching system, allowing reproducible HDX measurements between 50 ms and 5 min, with a 1 ms timescale resolution and temperature control (0–25°C) (Kish et al., 2019). The authors used the setup to uncover novel mechanistic details regarding the structural dynamics involved in the allosteric regulation of GlyP. They correlated structural changes in the activated (pSer14) and inhibited (glucose-6-phosphate bound) forms of the enzyme to functional regulation, determining that the 250s’ loop transitions from a disordered state to a more ordered conformation upon activation of the enzyme. A change in stability of the 280 s loop was also identified that provided the first direct evidence of allosteric regulation of substrate access to the active site.

**FIGURE 4:**
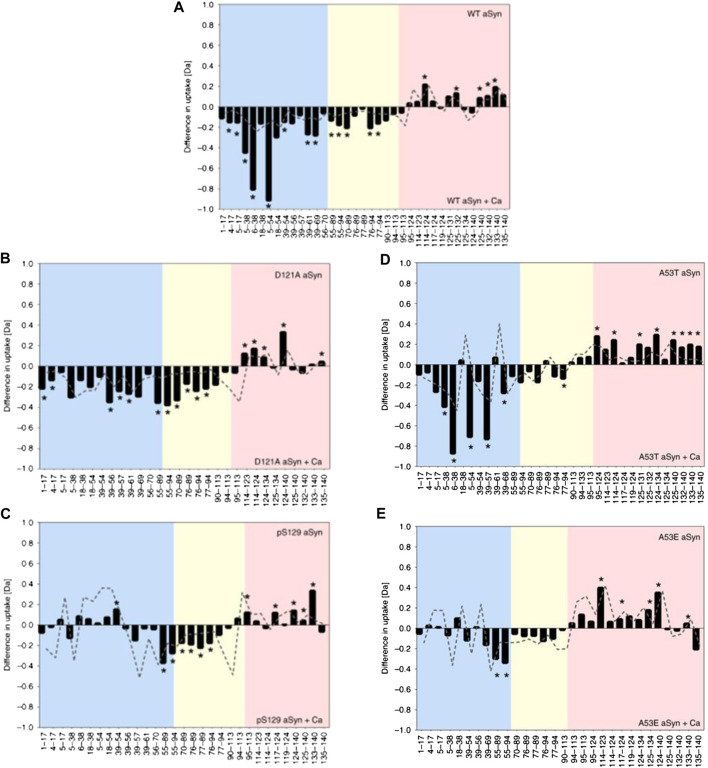
HDX-MS reveals differential responses of aSyn variants to calcium binding. Differential deuterium uptake between aSyn in the absence and presence of added calcium for WT aSyn **(A)**, variants D121A **(B)**, and pS129 **(C)** and familial mutations A53T **(D)** and A53E **(E)**. The N-terminus is colored blue, the NAC yellow, and C-terminus red. Negative values represent increased deuterium uptake in the calcium-bound state, correlating to increased solvent exposure and lower hydrogen bonding. Positive values represent decreased deuterium uptake in the presence of calcium. The start and end of each peptide is marked on the *x*-axis. Figure adapted from (Stephens et al., 2020).

Whilst proving to be highly beneficial, specialized msec fast HDX setups are not always required for analysis of IDPs. Stephens et al. (2020) used a commercially available HDX manager (Waters, USA) equipped with a sample handling robot (LEAP technologies, USA) to delineate how changes in the structural preferences of monomeric aSyn correlate with its propensity to aggregate. Solvent exposure of WT aSyn was compared with a phosphorylated version at residue S129 (pS129) and a mutant version (D121A). pS129 has an increased C-terminus negative charge and is commonly found in Lewy bodies but rarely in its soluble state, indicating increased aggregation propensity. D121A has a reduced C-terminal negative charge and a reduced aggregation propensity compared to the wildtype. The response of the constructs to calcium binding was compared, as calcium binds at the C-terminus, neutralizes negative charges, skews conformational preferences, and enhances the rate of aggregation. Upon calcium binding by WT aSyn, the rate of HDX becomes lower in the C-terminus (residues 95–140) and higher in the NAC (residues 61–95) and N-terminus (residue 1–61), indicating more compact and extended orientations, respectively (Figure 4A). The mutant D121A undergoes a similar change (Figure 4B), but for pS129 (Figure 4C), the N-terminus is much less solvent exposed, indicating a correlation between the solvent exposure of the N-terminal region with reduced aggregation propensity in the calcium-bound state. To further investigate the correlation between monomeric structure and aggregation propensity, the aggregation rates of familial aSyn mutants A30P, E46K, H50Q, G51D, A53T, and A53E were studied with ThT-based kinetic assays. It was determined that A53T, E46K, and H50Q aggregate faster than the WT protein, and the aggregation rate can be further enhanced by the addition of calcium. A30P, A53E, and G51D aggregate slower than WT aSyn and are insensitive to calcium or aggregate slower in its presence. A53T and A53E conformations were therefore probed via HDX, as the T and E mutations at position 53 have opposite effects on aggregation rates (increased rate and decreased rate, respectively). In the absence of calcium, no conformational differences could be identified between the mutants or between mutant and WT. In the presence of calcium, protection is observed at the C-terminus (as is identified with all other variants). A53T, like WT, has significant deprotection of N-terminus upon calcium binding (Figure 4D), indicating a localized unfolding event; whereas for A53E, there are very few significant differences at the NAC or N-terminus (Figure 4E). Therefore, perturbation in the N-terminal region upon calcium binding correlates with higher aggregation propensity. Despite much research into the aggregation behavior of aSyn, this method represents an important development in the ability to correlate IDP conformation with aggregation propensity.

## Chemical Crosslinking MASS SPECTROMETRY

Chemical crosslinking mass spectrometry (XL-MS) informs on the structure of proteins and protein complexes, by identifying residue pairs that are within close spatial proximity (Calabrese and Radford 2018; Schneider et al., 2018; Chavez and Bruce 2019; Iacobucci et al., 2020). These residue-level distance restraints are particularly useful in integrative structural studies, wherein information from a range of techniques is utilized to make structural predictions (Calabrese and Pukala 2013; Kahraman et al., 2013; Schmidt et al., 2013; Schmidt and Robinson 2014; Sinz 2014; Faini et al., 2016; Joseph et al., 2017). A typical XL-MS workflow involves incubation of the protein assembly of interest with a suitable crosslinking reagent that covalently links proximal residues targeted by the reactive groups of the crosslinker. The most common commercially available crosslinkers comprise two N-hydroxysuccinimide- (NHS-) ester functional groups, connected by a spacer arm, that primarily react with Lys side-chains and to a lesser extent of those of Ser, Thr, and Tyr (Kalkhof and Sinz 2008; Madler et al., 2009). Recent developments to increase the applicability of XL-MS to proteins with more diverse primary structures include crosslinking reagents that target carboxylic acids (hydrazides) (Leitner et al., 2014) and radical based methods (e.g., photoreactive diazirine and benzophenone) (Preston et al., 2014; Horne et al., 2018) that react nonspecifically with any sidechain. The spacer arm can also be decorated with functionalities to assist in identification of crosslinked peptides by semiautomated spectral assignment methods (by incorporating MS-cleavable groups) or facilitate enrichment (e.g., biotin, alkyne, or phosphonic acid) (Leitner et al., 2012; Gotze et al., 2015; Schmidt and Sinz 2017; Iacobucci et al., 2018; Yu and Huang 2018; Steigenberger et al., 2019). Enrichment is the key to XL-MS workflows when studying large, complex systems due to the inherent low abundance of XL peptides.

One of the challenges when using XL-MS reagents to study dynamic proteins/assemblies with multiple copopulated states is that the experimentally derived distance restraints constitute a snapshot of interresidue distances spanning the ensemble (Rappsilber 2011; Bonomi et al., 2017; Filella-Merce et al., 2020). In such cases, computational methods can be used to derive structural ensembles consistent with the data (Ding et al., 2017; Webb et al., 2018; Calabrese et al., 2020). Alternatively, the distance restraints from XL-MS can be mapped onto previously determined ensembles (Degiacomi et al., 2017; Bullock et al., 2018; Calabrese et al., 2020). For IDPs, untangling and modeling these conformational families using restraints from XL-MS, and indeed using data from any technique, remains challenging.

Several studies have used XL-MS as a tool to interrogate the architectures of IDPs that undergo pathogenic self-assembly in order to elucidate the structural/molecular determinants of amyloid formation. In one recent study, restraints derived from XL experiments were used in molecular dynamics simulations to derive model structures of monomeric tau (Popov et al., 2019). The models derived were globular in nature, whereas other evidence suggests that tau populates an ensemble of structures, including more elongated states (Mylonas et al., 2008; Narayanan et al., 2010; Elbaum-Garfinkle and Rhoades 2012; Mirbaha et al., 2018). This suggests that XL-MS may be overrepresenting the abundance of globular states in solution as has been observed for other flexible systems (Calabrese et al., 2020). A similar approach has also been used to derive a conformational ensemble for the protein aSyn. Again, compact conformations were visualized (Brodie et al., 2016; Brodie et al., 2019), which does not capture the breadth of the conformational ensemble, as demonstrated using other techniques (Nath et al., 2012; Schwalbe et al., 2014). These studies show that challenges remain in using XL-MS restraints to derive structural models that represent the overall ensembles of IDPs, especially in capturing the more elongated states adopted. Nevertheless, the study of these monomeric aggregation-prone proteins can be used as a starting point to understand their assembly mechanism and associated conformational conversions in more detail.

**FIGURE 5:**
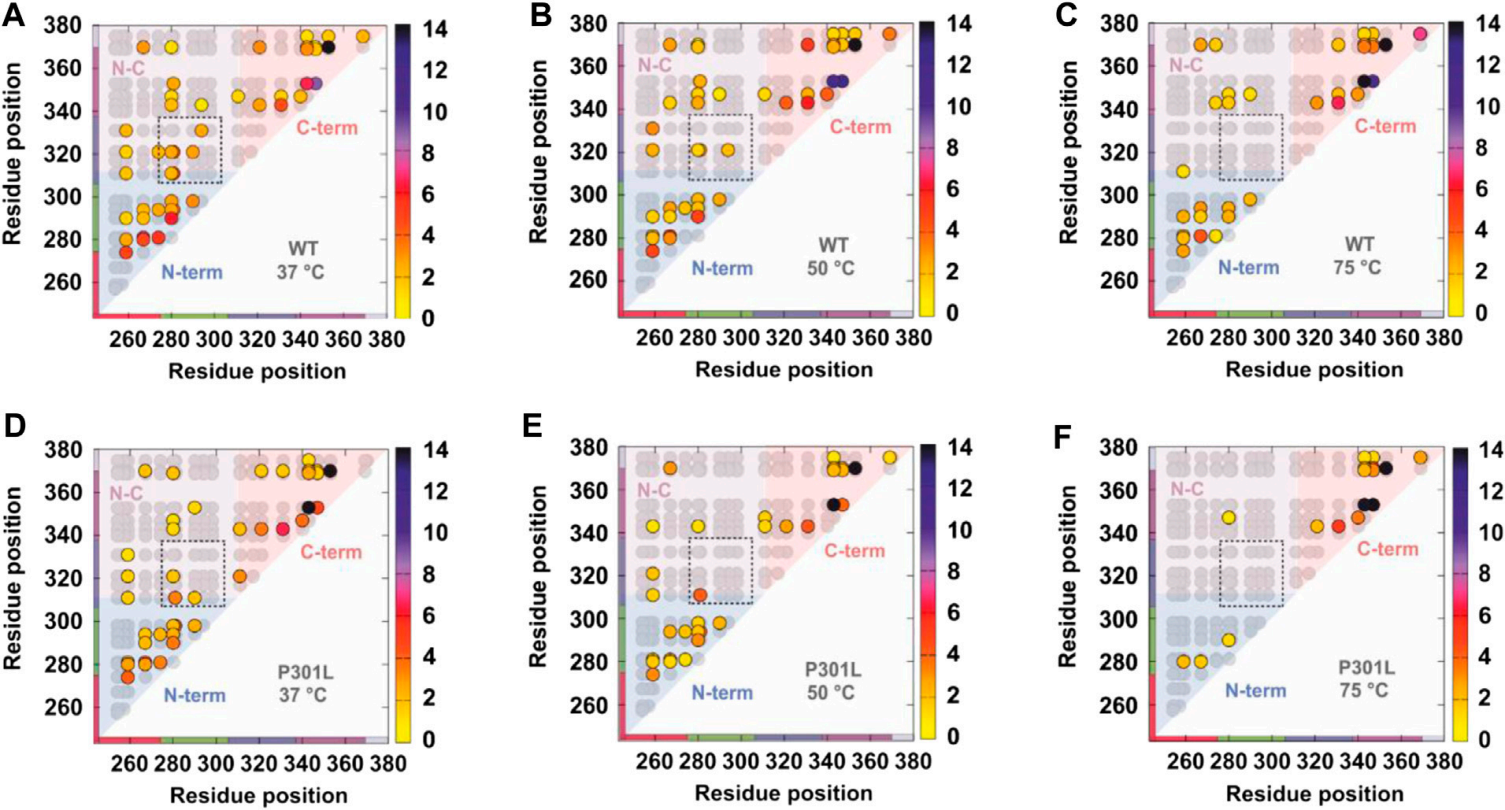
Comparative crosslinking of the tau repeat domain. Contact maps showing all theoretical crosslinks (Lys-Lys pairs) in the tau repeat domain (gray circles) and the detected crosslinks (colored circles). **(A–C)** Detected crosslinks in the wild type tau repeat domain at **(A)** 37°C, **(B)** 50°C, and **(C)** 75°C. **(D–F)** Detected crosslinks in the P301L tau repeat domain at **(A)** 37°C, **(B)** 50°C, and **(C)** 75°C. The detected crosslinks are colored according to the frequency by which they were observed—the scale is shown on the right hand side of each panel. The contact maps are shaded to denote crosslinks within the N-terminus (blue), C-terminus (red), and between the N- and C-termini (pink). Note that fewer long range contacts are observed with increasing temperature and that the P301L mutation promotes extended states (fewer long range crosslinks). Figure reproduced from (Chen et al., 2019).

Comparative XL has also been used to decode interregion contacts in tau and elucidate how mutations that are associated with enhanced aggregation kinetics alter the structure of the monomer (Chen et al., 2019). Disease-associated mutations known to influence tau aggregation have been shown to occur near the amyloid motif that drives self-assembly and is involved in key contacts in patient-derived tau amyloid fibrils (Chen et al., 2019). The authors studied the effect of the mutation P301L, a known familial mutation that is associated with increased neurodegeneration in model systems (Rizzu et al., 1999; Ramsden et al., 2005). Crosslinking experiments were performed using the tau repeat domain at three temperatures (37°, 50°, and 75°) to probe the relative thermostabilities of the wild type and P301L variant (Figure 5) (Chen et al., 2019). Contacts within the C-terminal and N-terminal domains were detected, as were crosslinks between the N and C termini (Figure 5) (Chen et al., 2019). Notably, P301L increased the susceptibility of tau to heat denaturation, as measured by fewer short and long-range XLs (Figure 5), suggesting that the mutation modulates the propensity of tau to adopt aggregation prone conformations that expose the key amyloid forming motif (Chen et al., 2019). These data combined with structural modeling and biophysical experiments suggested that P301L promotes the formation of extended tau conformations and implies that flanking regions surrounding key amyloid-forming motifs are keys to regulating tau aggregation propensity.

A similar comparative approach has been used to elucidate interresidue contacts between the N and C terminal domains of the cellular isoform of the prion protein (PrP^C^) (McDonald et al., 2019). When PrP^C^ undergoes conformational conversion to an infectious isoform (PrP^Sc^), it undergoes aggregation leading to toxic deposits associated with prion diseases (Ambadi Thody et al., 2018). PrP^C^ comprises a disordered N-terminal domain and a folded C-terminal domain whose structure has been solved (Riek et al., 1996; Zahn et al., 2000), but the determinants of the interaction between the two domains remained unknown (Spevacek et al., 2013; Evans et al., 2016; Wu et al., 2017). The function of PrP^C^ in healthy individuals also remains mysterious; however, it is known to bind divalent metal ions (Spevacek et al., 2013; Evans et al., 2016; Wu et al., 2017). XL-MS was used to elucidate interdomain contacts in murine PrP^C^, as well as two variants, one in which the central region of the protein was deleted (known to result in a neonatal lethal phenotype in mice (Li et al., 2007)) and the other which contained a single point mutation in the C-terminal domain (E199K) (the homologous murine mutation of the E200K mutation associated with familial Creutzfeldt–Jakob disease in humans) (Mead 2006). Crosslinks between the N- and C-terminal domains were detected in the wild type protein, and using quantitative methods, it was shown that Cu^2+^ binding triggers a conformational change in the protein, as the abundance of some crosslinked peptides pairs varied in the presence of Cu^2+^ (McDonald et al., 2019). Similarly, structural changes were detected between the wild type and two variants. These data were corroborated with those from paramagnetic relaxation enhancement (PRE) NMR experiments (Karamanos et al., 2015), and the combined data were used in MD simulations to generate models of PrP^C^.

Whilst XL-MS can provide residue level distance restraints, native MS can inform on the global properties of a protein/assembly without ensemble averaging. Thus, combining these two methods is becoming increasingly a commonplace for structural studies (Sinz et al., 2015). Such an approach was used to study the architecture of the p53 monomer and tetramer (Arlt et al., 2017). p53 is ca. 40% intrinsically disordered DNA binding protein that functions as a transcription factor (Laptenko et al., 2016), playing a crucial role in cancer prevention as a tumor suppressor (Hafner et al., 2019). Native MS using a mixture of heavy and light p53 suggested that the tetramer assembles as a dimer of dimers. Inter and intramolecular crosslinks in p53 were detected (Arlt et al., 2017) and compared with a model of p53 from a previous integrative study (Tidow et al., 2007). Many detected crosslinks were inconsistent with this structure, which led to the authors proposing that a structural reassignment of the assembly was necessary (Arlt et al., 2017). Native MS and XL-MS has also been used to study oligomerization of synaptobrevin-2 (Wittig et al., 2019), a membrane protein that is a constituent of the SNARE complex, which is involved in signal transmission in neurons (Takamori et al., 2006). The authors showed using crosslinking that in proteoliposomes, full-length synaptobrevin-2 was monomeric, while in detergent, low abundance oligomers could be captured by crosslinking (Wittig et al., 2019). A truncated version of synaptobrevin-2 had a higher oligomerization propensity than the wild type protein, but similar interaction interfaces were detected to the full length protein by XL-MS (Wittig et al., 2019). Native ion mobility-MS showed that the synaptobrevin-2 oligomers grew isotropically, consistent with them remaining dynamically disordered (Bleiholder et al., 2011).

## Footprinting MASS SPECTROMETRY

Chemical footprinting methods rely on treating a protein assembly with a chemical probe. The mass addition from the probe can be detected and quantified by MS to inform on the solvent accessibility of residues and how this changes with time or upon a binding event that is thought to perturb the structure (Wang and Chance 2017; Liu et al., 2020). Such methods have shown great promise to inform on the architecture of proteins and their assemblies and are now emerging as powerful tools to interrogate IDP structure, assembly, and interactions.

Hydroxyl radicals have been extensively used to study IDP structure. A number of methods have been developed to produce hydroxyl radicals (**˙**OH) to label surface-exposed residues, including using fast photochemical oxidation of proteins (FPOP) (Hambly and Gross 2005), synchrotron radiolysis of water (Wang and Chance 2011; Wang and Chance 2017), and atmospheric plasma jets (De Backer et al., 2018). The reactivity of residue sidechains toward hydroxyl radicals is a function of both its solvent accessibility (Huang et al., 2015), local environment (Cornwell et al., 2018), and intrinsic reactivity (Takamoto and Chance 2006; Xu and Chance 2007). The intrinsic reaction rates of different amino acids have been measured and span four orders of magnitude, with sulfur-containing (Cys and Met) and aromatic (Trp, Tyr, Phe, and His) residues being the most reactive (Takamoto and Chance 2006; Xu and Chance 2007). In such experiments, mass additions of 16 Da (addition of OH) are typically detected, but other modifications can also occur (Xu and Chance 2007) such as +14 Da modifications as a result of aldehyde/ketone formation. Quantification of the abundance of these modifications at either the peptide or residue level is typically achieved using LC-MS and bottom up proteomics methods.

**FIGURE 6:**
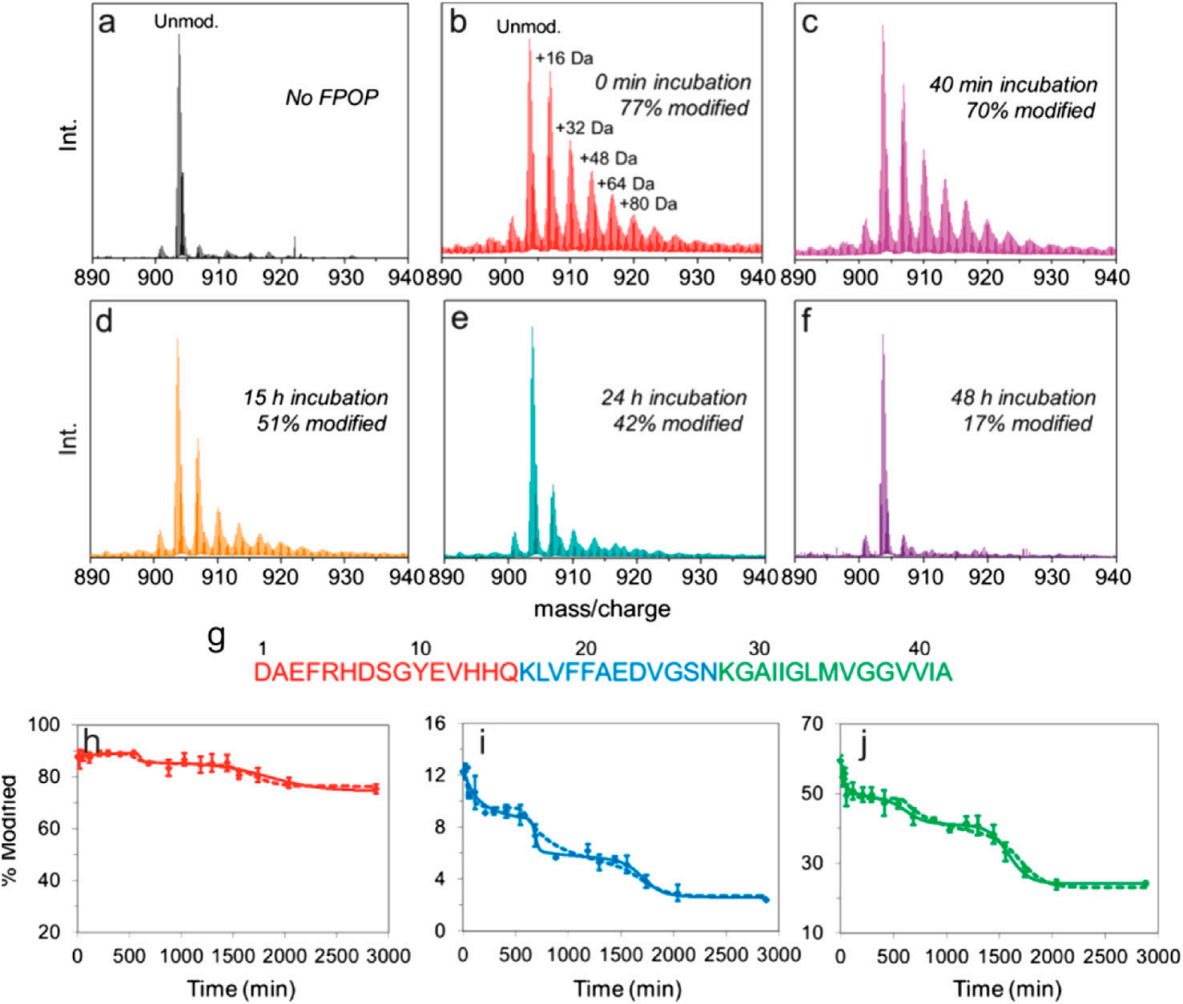
FPOP analysis to probe amyloid-*β* (1–42) amyloid assembly. **(A–F)** Intact mass spectra of amyloid-*β* (1–42) (5+ charge state) **(A)** not subjected to FPOP and after FPOP labeling at **(B)** 0 min, **(C)** 40 min, **(D)** 15 h, **(E)** 24 h, and **(F)** 48 h aggregation timepoints. **(G)** Sequence of amyloid-*β* (1–42) colored according to the three peptides produced after Lys-N digestion. **(H–J)** Extent of modification of the three Lys-N peptides of amyloid-*β* (1–42) as a function of aggregation time for the **(H)** N-terminal peptides, **(I)** central peptide, and **(J)** C-terminal peptide. The solid and dashed lines are simulated data derived using a simple kinetic model as outlined in (Li et al., 2016). Figure adapted with permission from (Li et al., 2016). Copyright: 2016 American Chemical Society.

In one recent example, Gross and coworkers used FPOP to pulse-label Aβ_1–42_ throughout its aggregation cycle (Li et al., 2016). During an FPOP experiment, low concentrations of H_2_O_2_ are added to a protein solution immediately prior to its irradiation at 248 nm with a pulsed laser (Hambly and Gross 2005). This generates **˙**OH that can react with solvent accessible sidechains (Hambly and Gross 2005). Scavengers (glutamine or histidine) are also added to the solution at concentrations that limit the lifetime of **˙**OH to less than ∼1 µs (i.e., the labeling pulse is faster than protein folding/unfolding) (Hambly and Gross 2005; Gau et al., 2013; Yan et al., 2014), although recent evidence suggests that radicals may be longer-lived (Vahidi and Konermann 2016). Dose-response experiments can also be performed by increasing/decreasing the scavenger concentration to alter the length of the labeling pulse (Niu et al., 2017). In this example, samples were taken at periodic intervals over a 48 h time course of Aβ_1–42_ aggregation. Analysis at the intact level revealed that, as expected, the extent of FPOP modifications decreased over time, as the protein formed higher order species (Figure 6). Remarkably, the data revealed several stepwise transitions in the extent of FPOP modification over time, consistent with a simplified kinetic model; wherein, monomeric species initially predominate that progressively oligomerize. Critical thresholds are reached throughout the aggregation time course that favor the formation of progressively larger oligomers, aggregates/protofibrils, and mature fibrils (Figure 6) (Li et al., 2016). The oxidative modifications on Aβ_1–42_ could be localized to the peptide and amino acid level to reveal key regions that are involved in structural transitions associated with aggregation (Figure 6). Pulsed HDX-MS has also been used to study Aβ_1–42_ aggregation under similar conditions, but this FPOP data revealed an additional conformational transition in the aggregation process that was not detected by HDX-MS (Zhang et al., 2013). Combined, this study demonstrates the power of FPOP to interrogate multistep self-assembly processes of IDPs, which will undoubtedly be a key tool going forward to temporally characterize, in residue-level detail, other assembly/aggregation mechanisms.

Data from oxidative footprinting by synchrotron radiolysis of water have also been used in integrative studies, combined with SAXS data to generate model IDP structures. To oxidatively label proteins by synchrotron radiolysis, a high energy synchrotron X-ray beam is used to generate **˙**OH by water photolysis (Takamoto and Chance 2006). This method has the advantage of being able to perform dose-response experiments by measuring the extent of oxidation as a function of X-ray exposure time (Takamoto and Chance 2006). From such data, a rate of footprinting can be measured on a per residue basis for comparison with the known intrinsic reactivity to derive a protection factor (Huang et al., 2015). There is a good correlation between protection factors derived from these experiments and the solvent accessibility of the residues in the protein structure (Huang et al., 2015). Specialized software called iSPOT (Huang et al., 2016) has been specifically developed to integrate SAXS and footprinting data in one modeling pipeline. This strategy was validated using protein assemblies with known structures (Popov et al., 2019), and has been recently used to study the intrinsically disordered N-terminal transactivation domain (NTD) of estrogen receptor-alpha (Peng et al., 2019). The iSPOT-derived structural models from SAXS and footprinting data revealed metastable long-range contacts in the IDP that may be essential for receptor function (Peng et al., 2019).

Ligand-directed footprinting strategies have also been developed to study the interactions of intrinsically disordered or unfolded proteins with folded protein counterparts. These protocols, described as ligand-footprint MS (LiF-MS) (Parker et al., 2019) and tag-transfer XL-MS (Horne et al., 2018), rely on installing a reactive diazirine moiety on the disordered/unfolded binding partner. After assembling the protein complex, UV irradiation produces a carbene that rapidly inserts into any nearby covalent bond (including with solvent), forming a crosslink at the interaction interface (Horne et al., 2018; Parker et al., 2019). Following cleavage of the crosslinker, a mass tag or fingerprint is left at the interaction site which can be identified by MS. The LiF-MS method has been validated by studying the interaction between SLiMs (short linear motifs) and mitogen-activated protein kinase (MAPK) (Parker et al., 2019). SLiMs are present in disordered regions of proteins that are involved in interactions with folded protein domains (Davey et al., 2015). Upon binding, the SLiM remains dynamically disordered, making structural characterization of these assemblies by crystallography challenging. The LiF-MS approach was validated using assemblies with available crystal structures and then applied to complexes with no high-resolution structures. Use of the data in computational docking revealed that the binding mode differs between MAPKs and that the complexes are malleable (Parker et al., 2019). A similar tag-transfer XL approach to study the interaction of dynamic proteins with folded proteins has also been developed. This method also exploits diazirine chemistry and uses MS to detect a mass tag left on the partner protein at the interaction site (Horne et al., 2018; Calabrese et al., 2020). This approach was validated using a high-affinity regulatory peptide-protein complex MCL1 with BID, with the detected crosslinks mapping to the binding cleft of MCL1 (Horne et al., 2018). This approach has been used to map interaction interfaces of unfolded outer membrane proteins with periplasmic chaperones Skp and SurA, revealing the primary sites on these chaperones which contact the unfolded clients (Horne et al., 2018; Calabrese et al., 2020).

One of the main challenges in using footprinting approaches to inform on protein structure is translating the detected chemical labeling intensity to a structural model (Schmidt et al., 2017; Aprahamian et al., 2018; Aprahamian and Lindert 2019), with recent evidence suggesting that rather than “solvent accessibility,” such experiments may report on a more nuanced parameter termed “chemical accessibility” (Ziemianowicz et al., 2020). Moreover, such methods also inherently report a snapshot of the conformational ensemble, which presents a significant methodological challenge for data interpretation that is exacerbated for IDPs which populate broad conformational landscapes.

## Conclusions and Outlook

Our understanding of the role of intrinsic disorder in biological mechanisms has dramatically expanded in recent years. Such advances could only be progressed by new approaches to interrogate protein structure and function, including developments in experimental methodologies, integrative methods, and computer simulations. Mass spectrometry, too, has seen a vast array of new tools developed that are specifically tailored, or ideally suited, to study IDPs. The power of subsecond HDX experiments is only now being realized (Rist et al., 2005; Liuni et al., 2010; Keppel et al., 2011; Yamamoto et al., 2011; Rob et al., 2012; Keppel and Weis 2013; Walters et al., 2013; Svejdal et al., 2019), and future developments in this area are essential to characterize IDP dynamics as they undergo motions on shorter timescales than folded proteins, for which experiments were first designed to interrogate. Similarly, rapid XL and covalent labeling chemistries are key to capturing structural information about dynamic ensembles over short timescales. Diazirines may be able to fulfill this role, as they react within nanoseconds (Admasu et al., 1998), but irradiation times of seconds to hours are still used (Horne et al., 2018).

It is now emerging that it is possible to generate drug-like small molecules that can target IDPs and/or their interactions with binding partners (Vassilev et al., 2004; Hammoudeh et al., 2009; Iconaru et al., 2015). In many cases, these small molecules do not function by high affinity binding, but rather by transient interactions that modulate the conformation ensemble. However, detecting structural perturbations by small molecules, and how this influences IDP/IDR function, remains challenging. In many cases, an integrative approach must be taken, which presents an ideal opportunity to apply MS techniques.

One exciting, growing area of MS capability lies in the ability to deduce structural information pertaining to proteins and their assemblies in situ, including in lysates, live cells, tissues, and organisms. The suite of structural MS methods spanning native MS, limited proteolysis, XL-MS, FPOP, and other covalent labeling techniques are all well suited to inform on IDP structure and assembly in situ. FPOP labeling can be performed in cell or in *C. elegans* (Espino et al., 2015; Espino and Jones 2019), and XL-MS pipelines have been developed to probe the architecture of protein assemblies directly in cells and tissues (Chavez et al., 2018; Götze et al., 2019; Mendes et al., 2019; O’Reilly et al., 2020). Rapid advances in experimental workflows and MS instrumentation have facilitated the use of structural MS to tackle this increased sample complexity.

Intriguingly, it has been shown using experiments to probe the thermal stability of proteins by limited proteolysis on a proteome-wide scale that only half of proteins had unfolding profiles consistent with them being intrinsically disordered (Leuenberger et al., 2017). This suggests that many predicted IDPs may be more rigid in situ, possibly as a result of ligand binding. Such an observation demonstrates the urgent unmet need for new tools to study IDP structure in-cell, with MS poized to play a leading role in this endeavor. Exciting opportunities include biotin labeling and biotin proximity tagging experiments that have been shown to selectively label IDPs/IDRs (Minde et al., 2020). One could envisage applications for such methods to probe disorder both in and out of cells, thereby validating assertions made about IDPs in situ. In addition, it is possible to generate native mass spectra of complexes directly from lysates, where proteins have been overexpressed (Vimer et al., 2020) or are present at endogenous levels (Skinner et al., 2017). New instrumentation and so-called native Omics workflows (Gault et al., 2020) have also been developed to facilitate multistage tandem mass spectrometry to enable ligand identification. One could envisage a role for these methods, in combination with top-down sequencing, to identify otherwise unknown modulators of IDP structure/function.

In recent years, developments in structural MS methods have been instrumental in advancing our understanding of protein behavior, shedding light on protein conformations and interactions. Now that the importance of IDPs in health and disease is becoming increasingly apparent and MS based methods tailored to study IDPs are being realized, structural MS is set to remain integral in the quest to decipher IDP structure and function, both *in vitro* and in cell, uncovering new insights into the role of IDPs in biological mechanisms.

## References

[B1] Acharya S. Safaie B. M. Wongkongkathep P. Ivanova M. I. Attar A. Klärner F. G. (2014). Molecular basis for preventing α-synuclein aggregation by a molecular tweezer. J. Biol. Chem. 289 (15), 10727–10737. 10.1074/jbc.M113.524520 24567327 PMC4036189

[B2] Admasu A. Gudmundsdóttir A. D. Platz M. S. Watt D. S. Kwiatkowski S. Crocker P. J. (1998). A laser flash photolysis study of p-tolyl(trifluoromethyl)carbene. J. Chem. Soc. Perkin Trans. 2 (5), 1093–1100. 10.1039/a707586c

[B3] Ahmadi S. Zhu S. Sharma R. Wilson D. J. Kraatz H. B. (2019). Interaction of metal ions with tau protein. The case for a metal-mediated tau aggregation. J. Inorg. Biochem. 194, 44–51. 10.1016/j.jinorgbio.2019.02.007 30826589

[B4] Al-Naqshabandi M. A. Weis D. D. (2017). Quantifying protection in disordered proteins using millisecond hydrogen exchange-mass spectrometry and peptic reference peptides. Biochemistry 56 (31), 4064–4072. 10.1021/acs.biochem.6b01312 28675294

[B5] Ambadi Thody S. Mathew M. K. Udgaonkar J. B. (2018). Mechanism of aggregation and membrane interactions of mammalian prion protein. Biochim. Biophys. Acta - Biomembranes 1860 (9), 1927–1935. 10.1016/j.bbamem.2018.02.031 29514050

[B6] Aprahamian M. L. Chea E. E. Jones L. M. Lindert S. (2018). Rosetta protein structure prediction from hydroxyl radical protein footprinting mass spectrometry data. Anal. Chem. 90 (12), 7721–7729. 10.1021/acs.analchem.8b01624 29874044 PMC6008241

[B7] Aprahamian M. L. Lindert S. (2019). Utility of covalent labeling mass spectrometry data in protein structure prediction with rosetta. J. Chem. Theor. Comput 15 (5), 3410–3424. 10.1021/acs.jctc.9b00101 PMC6520167 30946594

[B8] Arlt C. Flegler V. Ihling C. H. Schäfer M. Thondorf I. Sinz A. (2017). An integrated mass spectrometry based approach to probe the structure of the full-length wild-type tetrameric p53 tumor suppressor. Angew. Chem. Int. Ed. Engl. 56 (1), 275–279. 10.1002/anie.201609826 27897373

[B9] Arya S. Claud S. L. Cantrell K. L. Bowers M. T. (2019). Catalytic prion-like cross-talk between a key alzheimer's disease tau-fragment R3 and the type 2 diabetes peptide IAPP. ACS Chem. Neurosci. 10 (11), 4757–4765. 10.1021/acschemneuro.9b00516 31642657

[B10] Baba M. Nakajo S. Tu P. H. Tomita T. Nakaya K. Lee V. M. (1998). Aggregation of alpha-synuclein in Lewy bodies of sporadic Parkinson's disease and dementia with Lewy bodies. Am. J. Pathol. 152 (4), 879–884. 9546347 PMC1858234

[B11] Baker C. M. Best R. B. (2014). Insights into the binding of intrinsically disordered proteins from molecular dynamics simulation. Wires Comput. Mol. Sci. 4 (3), 182–198. 10.1002/wcms.1167 PMC8336759 34354764

[B12] Banani S. F. Lee H. O. Hyman A. A. Rosen M. K. (2017). Biomolecular condensates: organizers of cellular biochemistry. Nat. Rev. Mol. Cell Biol 18 (5), 285–298. 10.1038/nrm.2017.7 28225081 PMC7434221

[B13] Beveridge R. Chappuis Q. Macphee C. Barran P. (2013). Mass spectrometry methods for intrinsically disordered proteins. Analyst 138 (1), 32–42. 10.1039/c2an35665a 23108160

[B14] Beveridge R. Covill S. Pacholarz K. J. Kalapothakis J. M. MacPhee C. E. Barran P. E. (2014). A mass-spectrometry-based framework to define the extent of disorder in proteins. Anal. Chem. 86 (22), 10979–10991. 10.1021/ac5027435 25353392

[B15] Beveridge R. Kessler D. Rumpel K. Ettmayer P. Meinhart A. Clausen T. (2020). Native mass spectrometry can effectively predict PROTAC efficacy. ACS Cent. Sci. 6 (7), 1223–1230. 10.1021/acscentsci.0c00049 32724856 PMC7379389

[B16] Beveridge R. Migas L. G. Das R. K. Pappu R. V. Kriwacki R. W. Barran P. E. (2019a). Ion mobility mass spectrometry uncovers the impact of the patterning of oppositely charged residues on the conformational distributions of intrinsically disordered proteins. J. Am. Chem. Soc. 141 (12), 4908–4918. 10.1021/jacs.8b13483 30823702 PMC6488185

[B17] Beveridge R. Migas L. G. Kriwacki R. W. Barran P. E. (2019b). Ion mobility mass spectrometry measures the conformational landscape of p27 and its domains and how this is modulated upon interaction with Cdk2/cyclin A. Angew. Chem. Int. Ed. Engl. 58 (10), 3114–3118. 10.1002/anie.201812697 30570821 PMC7122115

[B18] Beveridge R. Migas L. G. Payne K. A. Scrutton N. S. Leys D. Barran P. E. (2016). Mass spectrometry locates local and allosteric conformational changes that occur on cofactor binding. Nat. Commun. 7, 12163. 10.1038/ncomms12163 27418477 PMC4947166

[B19] Bleiholder C. Dupuis N. F. Wyttenbach T. Bowers M. T. (2011). Ion mobility-mass spectrometry reveals a conformational conversion from random assembly to β-sheet in amyloid fibril formation. Nat. Chem. 3 (2), 172–177. 10.1038/nchem.945 21258392 PMC3073516

[B20] Boehr D. D. Nussinov R. Wright P. E. (2009). The role of dynamic conformational ensembles in biomolecular recognition. Nat. Chem. Biol. 5 (11), 789–796. 10.1038/nchembio.232 19841628 PMC2916928

[B21] Bonomi M. Heller G. T. Camilloni C. Vendruscolo M. (2017). Principles of protein structural ensemble determination. Curr. Opin. Struct. Biol. 42, 106–116. 10.1016/j.sbi.2016.12.004 28063280

[B22] Breuker K. McLafferty F. W. (2008). Stepwise evolution of protein native structure with electrospray into the gas phase, 10^−12^ to 10^2^ s. Proc. Natl. Acad. Sci. USA 105 (47), 18145–18152. 10.1073/pnas.0807005105 19033474 PMC2587555

[B23] Brodie N. I. Petrotchenko E. V. Borchers C. H. (2016). The novel isotopically coded short-range photo-reactive crosslinker 2,4,6-triazido-1,3,5-triazine (TATA) for studying protein structures. J. Proteomics 149, 69–76. 10.1016/j.jprot.2016.02.024 26931439

[B24] Brodie N. I. Popov K. I. Petrotchenko E. V. Dokholyan N. V. Borchers C. H. (2019). Conformational ensemble of native α-synuclein in solution as determined by short-distance crosslinking constraint-guided discrete molecular dynamics simulations. PLoS Comput. Biol. 15 (3), e1006859. 10.1371/journal.pcbi.1006859 30917118 PMC6453469

[B25] Bullock J. M. A. Thalassinos K. Topf M. (2018). Jwalk and MNXL web server: model validation using restraints from crosslinking mass spectrometry. Bioinformatics 34 (20), 3584–3585. 10.1093/bioinformatics/bty366 29741581 PMC6184817

[B26] Bush M. F. Campuzano I. D. Robinson C. V. (2012). Ion mobility mass spectrometry of peptide ions: effects of drift gas and calibration strategies. Anal. Chem. 84 (16), 7124–7130. 10.1021/ac3014498 22845859

[B27] Calabrese A. N. Radford S. E. (2018). Mass spectrometry-enabled structural biology of membrane proteins. Methods 147, 187–205. 10.1016/j.ymeth.2018.02.020 29510247

[B28] Calabrese A. N. Pukala T. L. (2013). Chemical cross-linking and mass spectrometry for the structural analysis of protein assemblies. Aust. J. Chem. 66 (7), 749–759. 10.1071/ch13164

[B29] Calabrese A. N. Schiffrin B. Watson M. Karamanos T. K. Walko M. Humes J. R. (2020). Inter-domain dynamics in the chaperone SurA and multi-site binding to its unfolded outer membrane protein clients. Nat. Comm. 11, 2155. 10.1038/s41467-020-15702-1 PMC7195389 32358557

[B30] Chavez J. D. Bruce J. E. (2019). Chemical cross-linking with mass spectrometry: a tool for systems structural biology. Curr. Opin. Chem. Biol. 48, 8–18. 10.1016/j.cbpa.2018.08.006 30172868 PMC6382564

[B31] Chavez J. D. Lee C. F. Caudal A. Keller A. Tian R. Bruce J. E. (2018). Chemical crosslinking mass spectrometry analysis of protein conformations and supercomplexes in heart tissue. Cell Syst 6 (1), 136–141. 10.1016/j.cels.2017.10.017 29199018 PMC5799023

[B32] Chen D. Drombosky K. W. Hou Z. Sari L. Kashmer O. M. Ryder B. D. (2019). Tau local structure shields an amyloid-forming motif and controls aggregation propensity. Nat. Commun. 10 (1), 2493. 10.1038/s41467-019-10355-1 31175300 PMC6555816

[B33] Chen S. H. Chen L. Russell D. H. (2014). Metal-induced conformational changes of human metallothionein-2A: a combined theoretical and experimental study of metal-free and partially metalated intermediates. J. Am. Chem. Soc. 136 (26), 9499–9508. 10.1021/ja5047878 24918957

[B34] Cleveland D. W. Hwo S. Y. Kirschner M. W. (1977). Physical and chemical properties of purified tau factor and the role of tau in microtubule assembly. J. Mol. Biol. 116 (2), 227–247. 10.1016/0022-2836(77)90214-5 146092

[B35] Cornwell O. Radford S. E. Ashcroft A. E. Ault J. R. (2018). Comparing hydrogen deuterium exchange and fast photochemical oxidation of proteins: a structural characterisation of wild-type and dn6 β2-microglobulin. J. Am. Soc. Mass Spectrom. 29 (12), 2413–2426. 10.1007/s13361-018-2067-y 30267362 PMC6276068

[B36] D'Urzo A. Konijnenberg A. Rossetti G. Habchi J. Li J. Carloni P. (2015). Molecular basis for structural heterogeneity of an intrinsically disordered protein bound to a partner by combined ESI-IM-MS and modeling. J. Am. Soc. Mass Spectrom. 26 (3), 472–481. 10.1007/s13361-014-1048-z 25510932

[B37] Das R. K. Huang Y. Phillips A. H. Kriwacki R. W. Pappu R. V. (2016). Cryptic sequence features within the disordered protein p27^Kip1^ regulate cell cycle signaling. Proc. Natl. Acad. Sci. USA 113 (20), 5616–5621. 10.1073/pnas.1516277113 27140628 PMC4878473

[B38] Das R. K. Pappu R. V. (2013). Conformations of intrinsically disordered proteins are influenced by linear sequence distributions of oppositely charged residues. Proc. Natl. Acad. Sci. USA 110 (33), 13392–13397. 10.1073/pnas.1304749110 23901099 PMC3746876

[B39] Davey N. E. Cyert M. S. Moses A. M. (2015). Short linear motifs - ex nihilo evolution of protein regulation. Cell Commun Signal 13 (1), 43. 10.1186/s12964-015-0120-z 26589632 PMC4654906

[B40] De Backer J. Razzokov J. Hammerschmid D. Mensch C. Hafideddine Z. Kumar N. (2018). The effect of reactive oxygen and nitrogen species on the structure of cytoglobin: a potential tumor suppressor. Redox Biol. 19, 1–10. 10.1016/j.redox.2018.07.019 30081385 PMC6084017

[B41] Degiacomi M. T. Schmidt C. Baldwin A. J. Benesch J. L. P. (2017). Accommodating protein dynamics in the modeling of chemical crosslinks. Structure 25 (11), 1751–e5. 10.1016/j.str.2017.08.015 28966018

[B42] Demarest S. J. Martinez-Yamout M. Chung J. Chen H. Xu W. Dyson H. J. (2002). Mutual synergistic folding in recruitment of CBP/p300 by p160 nuclear receptor coactivators. Nature 415 (6871), 549–553. 10.1038/415549a 11823864

[B43] Ding Y. H. Gong Z. Dong X. Liu K. Liu Z. Liu C. (2017). Modeling protein excited-state structures from "Over-length" chemical cross-links. J. Biol. Chem. 292 (4), 1187–1196. 10.1074/jbc.M116.761841 27994050 PMC5270465

[B44] Dong S. Wagner N. D. Russell D. H. (2018). Collision-induced unfolding of partially metalated metallothionein-2A: tracking unfolding reactions of gas-phase ions. Anal. Chem. 90 (20), 11856–11862. 10.1021/acs.analchem.8b01622 30221929 PMC6463490

[B45] Donnelly D. P. Rawlins C. M. DeHart C. J. Fornelli L. Schachner L. F. Lin Z. (2019). Best practices and benchmarks for intact protein analysis for top-down mass spectrometry. Nat. Methods 16 (7), 587–594. 10.1038/s41592-019-0457-0 31249407 PMC6719561

[B46] Downey M. A. Giammona M. J. Lang C. A. Buratto S. K. Singh A. Bowers M. T. (2019). Inhibiting and remodeling toxic amyloid-beta oligomer formation using a computationally designed drug molecule that targets alzheimer's disease. J. Am. Soc. Mass Spectrom. 30 (1), 85–93. 10.1007/s13361-018-1975-1 29713966 PMC6258352

[B47] Economou N. J. Giammona M. J. Do T. D. Zheng X. Teplow D. B. Buratto S. K. (2016). Amyloid β-protein assembly and alzheimer's disease: dodecamers of Aβ42, but not of Aβ40, seed fibril formation. J. Am. Chem. Soc. 138 (6), 1772–1775. 10.1021/jacs.5b11913 26839237 PMC4849547

[B48] Elbaum-Garfinkle S. Rhoades E. (2012). Identification of an aggregation-prone structure of tau. J. Am. Chem. Soc. 134 (40), 16607–16613. 10.1021/ja305206m 22998648 PMC3477793

[B49] Eldrid C. Ujma J. Kalfas S. Tomczyk N. Giles K. Morris M. (2019). Gas phase stability of protein ions in a cyclic ion mobility spectrometry traveling wave device. Anal. Chem. 91 (12), 7554–7561. 10.1021/acs.analchem.8b05641 31117399 PMC7006968

[B50] Eldrid C. Ujma J. Britt H. Cragnolini T. Kalfas S. Cooper-Shepherd D. (2020). Cyclic ion mobility – collision activation experiments elucidate protein behaviour in the gas-phase. 10.26434/chemrxiv.11687100 PMC8172447 34006100

[B51] Eschmann N. A. Do T. D. LaPointe N. E. Shea J. E. Feinstein S. C. Bowers M. T. (2015). Tau aggregation propensity engrained in its solution state. J. Phys. Chem. B 119 (45), 14421–14432. 10.1021/acs.jpcb.5b08092 26484390 PMC4645975

[B52] Espino J. A. Jones L. M. (2019). Illuminating biological interactions with *in Vivo* protein footprinting. Anal. Chem. 91 (10), 6577–6584. 10.1021/acs.analchem.9b00244 31025855 PMC6533598

[B53] Espino J. A. Mali V. S. Jones L. M. (2015). In cell footprinting coupled with mass spectrometry for the structural analysis of proteins in live cells. Anal. Chem. 87 (15), 7971–7978. 10.1021/acs.analchem.5b01888 26146849

[B54] Evans E. G. Pushie M. J. Markham K. A. Lee H. W. Millhauser G. L. (2016). Interaction between prion protein's copper-bound octarepeat domain and a charged C-terminal pocket suggests a mechanism for N-terminal regulation. Structure 24 (7), 1057–1067. 10.1016/j.str.2016.04.017 27265848 PMC4938727

[B55] Eyers C. E. Vonderach M. Ferries S. Jeacock K. Eyers P. A. (2018). Understanding protein-drug interactions using ion mobility-mass spectrometry. Curr. Opin. Chem. Biol. 42, 167–176. 10.1016/j.cbpa.2017.12.013 29331721

[B56] Faini M. Stengel F. Aebersold R. (2016). The evolving contribution of mass spectrometry to integrative structural biology. J. Am. Soc. Mass Spectrom. 27 (6), 966–974. 10.1007/s13361-016-1382-4 27056566 PMC4867889

[B57] Ferrer I. López-González I. Carmona M. Arregui L. Dalfó E. Torrejón-Escribano B. (2014). Glial and neuronal tau pathology in tauopathies: characterization of disease-specific phenotypes and tau pathology progression. J. Neuropathol. Exp. Neurol. 73 (1), 81–97. 10.1097/NEN.0000000000000030 24335532

[B58] Filella-Merce I. Bardiaux B. Nilges M. Bouvier G. (2020). Quantitative structural interpretation of protein crosslinks. Structure 28 (1), 75–e4. 10.1016/j.str.2019.10.018 31753619

[B59] Fitzpatrick A. W. P. Falcon B. He S. Murzin A. G. Murshudov G. Garringer H. J. (2017). Cryo-EM structures of tau filaments from Alzheimer’s disease. Nature 547 (7662), 185–190. 10.1038/nature23002 28678775 PMC5552202

[B60] Fraga H. Pujols J. Gil-Garcia M. Roque A. Bernardo-Seisdedos G. Santambrogio C. (2017). Disulfide driven folding for a conditionally disordered protein. Sci. Rep. 7 (1), 16994. 10.1038/s41598-017-17259-4 29208936 PMC5717278

[B61] Gabelica V. Marklund E. (2018). Fundamentals of ion mobility spectrometry. Curr. Opin. Chem. Biol. 42, 51–59. 10.1016/j.cbpa.2017.10.022 29154177

[B62] Ganguly P. Do T. D. Larini L. LaPointe N. E. Sercel A. J. Shade M. F. (2015). Tau assembly: the dominant role of PHF6 (VQIVYK) in microtubule binding region repeat R3. J. Phys. Chem. B 119 (13), 4582–4593. 10.1021/acs.jpcb.5b00175 25775228 PMC4428543

[B63] Garabedian A. Bolufer A. Leng F. Fernandez-Lima F. (2018). Peptide sequence influence on the conformational dynamics and DNA binding of the intrinsically disordered AT-hook 3 peptide. Sci. Rep. 8 (1), 10783. 10.1038/s41598-018-28956-z 30018295 PMC6050235

[B64] Gau B. C. Chen J. Gross M. L. (2013). Fast photochemical oxidation of proteins for comparing solvent-accessibility changes accompanying protein folding: data processing and application to barstar. Biochim. Biophys. Acta 1834, 1230–1238. 10.1016/j.bbapap.2013.02.023 23485913 PMC3663899

[B65] Gault J. Liko I. Landreh M. Shutin D. Bolla J. R. Jefferies D. (2020). Combining native and 'omics' mass spectrometry to identify endogenous ligands bound to membrane proteins. Nat. Methods 17 (5), 505–508. 10.1038/s41592-020-0821-0 32371966 PMC7332344

[B66] Giles K. Ujma J. Wildgoose J. Pringle S. Richardson K. Langridge D. (2019). A cyclic ion mobility-mass spectrometry system. Anal. Chem. 91 (13), 8564–8573. 10.1021/acs.analchem.9b01838 31141659

[B67] Goswami D. Devarakonda S. Chalmers M. J. Pascal B. D. Spiegelman B. M. Griffin P. R. (2013). Time window expansion for HDX analysis of an intrinsically disordered protein. J. Am. Soc. Mass Spectrom. 24 (10), 1584–1592. 10.1007/s13361-013-0669-y 23884631 PMC3773365

[B68] Götze M. Pettelkau J. Fritzsche R. Ihling C. H. Schäfer M. A. Sinz A. (2015). Automated assignment of MS/MS cleavable cross-links in protein 3D-structure analysis. J. Am. Soc. Mass Spectrom. 26 (1), 83–97. 10.1007/s13361-014-1001-1 25261217

[B69] Götze M. Iacobucci C. Ihling C. H. Sinz A. (2019). A simple cross-linking/mass spectrometry workflow for studying system-wide protein interactions. Anal. Chem. 91 (15), 10236–10244. 10.1021/acs.analchem.9b02372 31283178

[B70] Hafner A. Bulyk M. L. Jambhekar A. Lahav G. (2019). The multiple mechanisms that regulate p53 activity and cell fate. Nat. Rev. Mol. Cell Biol 20 (4), 199–210. 10.1038/s41580-019-0110-x 30824861

[B71] Hagiwara Y. Sieverling L. Hanif F. Anton J. Dickinson E. R. Bui T. T. (2016). Consequences of point mutations in melanoma-associated antigen 4 (MAGE-A4) protein: insights from structural and biophysical studies. Sci. Rep. 6 (1), 25182. 10.1038/srep25182 27121989 PMC4848555

[B72] Hambly D. M. Gross M. L. (2005). Laser flash photolysis of hydrogen peroxide to oxidize protein solvent-accessible residues on the microsecond timescale. J. Am. Soc. Mass Spectrom. 16 (12), 2057–2063. 10.1016/j.jasms.2005.09.008 16263307

[B73] Hamdi K. Salladini E. O'Brien D. P. Brier S. Chenal A. Yacoubi I. (2017). Structural disorder and induced folding within two cereal, ABA stress and ripening (ASR) proteins. Sci. Rep. 7 (1), 15544. 10.1038/s41598-017-15299-4 29138428 PMC5686140

[B74] Hammoudeh D. I. Follis A. V. Prochownik E. V. Metallo S. J. (2009). Multiple independent binding sites for small-molecule inhibitors on the oncoprotein c-myc. J. Am. Chem. Soc. 131 (21), 7390–7401. 10.1021/ja900616b 19432426

[B75] Hollstein M. Sidransky D. Vogelstein B. Harris C. (1991). p53 mutations in human cancers. Science 253 (5015), 49–53. 10.1126/science.1905840 1905840

[B76] Horne J. E. Walko M. Calabrese A. N. Levenstein M. A. Brockwell D. J. Kapur N. (2018). Rapid mapping of protein interactions using tag-transfer photocrosslinkers. Angew. Chem. Int. Ed. Engl. 57 (51), 16688–16692. 10.1002/anie.201809149 30393918 PMC6348423

[B77] Huang W. Ravikumar K. M. Chance M. R. Yang M. R. S. (2015). Quantitative mapping of protein structure by hydroxyl radical footprinting-mediated structural mass spectrometry: a protection factor Analysis. Biophys. J. 108 (1), 107–115. 10.1016/j.bpj.2014.11.013 25564857 PMC4286602

[B78] Huang W. Ravikumar K. M. Parisien M. Yang S. (2016). Theoretical modeling of multiprotein complexes by iSPOT: integration of small-angle X-ray scattering, hydroxyl radical footprinting, and computational docking. J. Struct. Biol. 196 (3), 340–349. 10.1016/j.jsb.2016.08.001 27496803 PMC5118146

[B79] Iacobucci C. Götze M. Ihling C. H. Piotrowski C. Arlt C. Schäfer M. (2018). A cross-linking/mass spectrometry workflow based on MS-cleavable cross-linkers and the MeroX software for studying protein structures and protein-protein interactions. Nat. Protoc. 13 (12), 2864–2889. 10.1038/s41596-018-0068-8 30382245

[B80] Iacobucci C. Götze M. Sinz A. (2020). Cross-linking/mass spectrometry to get a closer view on protein interaction networks. Curr. Opin. Biotechnol. 63, 48–53. 10.1016/j.copbio.2019.12.009 31891863

[B81] Iconaru L. I. Ban D. Bharatham K. Ramanathan A. Zhang W. Shelat A. A. (2015). Discovery of small molecules that inhibit the disordered protein, p27(Kip1). Sci. Rep. 5 (1), 15686. 10.1038/srep15686 26507530 PMC4623604

[B82] Ilitchev A. I. Giammona M. J. Olivas C. Claud S. L. Lazar Cantrell K. L. Wu C. (2018). Hetero-oligomeric amyloid assembly and mechanism: prion fragment PrP(106-126) Catalyzes the islet amyloid polypeptide β-hairpin. J. Am. Chem. Soc. 140 (30), 9685–9695. 10.1021/jacs.8b05925 29989407

[B83] Illes-Toth E. Ramos M. R. Cappai R. Dalton C. David P. (2015). Distinct higher-order α-synuclein oligomers induce intracellular aggregation. Biochem. J. 468 (3), 485–493. 10.1042/BJ20150159 25851527

[B84] Irvine G. B. El-Agnaf O. M. Shankar G. M. Walsh D. M. (2008). Protein aggregation in the brain: the molecular basis for Alzheimer's and Parkinson's diseases. Mol. Med. 14 (7), 451–464. 10.2119/2007-00100.Irvine 18368143 PMC2274891

[B85] Jebarupa B. Muralidharan M. Srinivasu B. Y. Mandal A. K. Mitra G. (2018). Effect of altered solution conditions on tau conformational dynamics: plausible implication on order propensity and aggregation. Biochim. Biophys. Acta - Proteins Proteomics 1866 (5), 668–679. 10.1016/j.bbapap.2018.04.004 29630971

[B86] Jensen M. R. Ruigrok R. W. Blackledge M. (2013). Describing intrinsically disordered proteins at atomic resolution by NMR. Curr. Opin. Struct. Biol. 23 (3), 426–435. 10.1016/j.sbi.2013.02.007 23545493

[B87] Joseph A. P. Polles G. Alber F. Topf M. (2017). Integrative modelling of cellular assemblies. Curr. Opin. Struct. Biol. 46, 102–109. 10.1016/j.sbi.2017.07.001 28735107 PMC5683902

[B88] Jurneczko E. Barran P. E. (2011). How useful is ion mobility mass spectrometry for structural biology? The relationship between protein crystal structures and their collision cross sections in the gas phase. Analyst 136 (1), 20–28. 10.1039/c0an00373e 20820495

[B89] Kahraman A. Herzog F. Leitner A. Rosenberger G. Aebersold R. Malmström L. (2013). Cross-link guided molecular modeling with ROSETTA. PLoS One 8 (9), e73411. 10.1371/journal.pone.0073411 24069194 PMC3775805

[B90] Kalkhof S. Sinz A. (2008). Chances and pitfalls of chemical cross-linking with amine-reactive N-hydroxysuccinimide esters. Anal. Bioanal. Chem. 392 (1–2), 305–312. 10.1007/s00216-008-2231-5 18724398

[B91] Kaltashov I. A. Mohimen A. (2005). Estimates of protein surface areas in solution by electrospray ionization mass spectrometry. Anal. Chem. 77 (16), 5370–5379. 10.1021/ac050511+ 16097782 PMC2631554

[B92] Karamanos T. K. Kalverda A. P. Thompson G. S. Radford S. E. (2015). Mechanisms of amyloid formation revealed by solution NMR. Prog. Nucl. Magn. Reson. Spectrosc. 88–89, 86–104. 10.1016/j.pnmrs.2015.05.002 PMC4568309 26282197

[B93] Keppel T. R. Howard B. A. Weis D. D. (2011). Mapping unstructured regions and synergistic folding in intrinsically disordered proteins with amide H/D exchange mass spectrometry. Biochemistry 50 (40), 8722–8732. 10.1021/bi200875p 21894929

[B94] Keppel T. R. Weis D. D. (2013). Analysis of disordered proteins using a simple apparatus for millisecond quench-flow H/D exchange. Anal. Chem. 85 (10), 5161–5168. 10.1021/ac4004979 23586525

[B95] Keppel T. R. Weis D. D. (2015). Mapping residual structure in intrinsically disordered proteins at residue resolution using millisecond hydrogen/deuterium exchange and residue averaging. J. Am. Soc. Mass Spectrom. 26 (4), 547–554. 10.1007/s13361-014-1033-6 25481641

[B96] Kikhney A. G. Svergun D. I. (2015). A practical guide to small angle X-ray scattering (SAXS) of flexible and intrinsically disordered proteins. FEBS Lett. 589 (19), 2570–2577. 10.1016/j.febslet.2015.08.027 26320411

[B97] Kish M. Smith V. Subramanian S. Vollmer F. Lethbridge N. Cole L. (2019). Allosteric regulation of glycogen phosphorylase solution phase structural dynamics at high spatial resolution. 10.1101/654665 PMC10116597 36989206

[B98] Kjaergaard M. Brander S. Poulsen F. M. (2011). Random coil chemical shift for intrinsically disordered proteins: effects of temperature and pH. J. Biomol. NMR 49 (2), 139–149. 10.1007/s10858-011-9472-x 21234644

[B99] Konijnenberg A. Ranica S. Narkiewicz J. Legname G. Grandori R. Sobott F. (2016). Opposite structural effects of epigallocatechin-3-gallate and dopamine binding to α-synuclein. Anal. Chem. 88 (17), 8468–8475. 10.1021/acs.analchem.6b00731 27467405

[B100] Lanucara F. Holman S. W. Gray C. J. Eyers C. E. (2014). The power of ion mobility-mass spectrometry for structural characterization and the study of conformational dynamics. Nat. Chem. 6 (4), 281–294. 10.1038/nchem.1889 24651194

[B101] Laptenko O. Tong D. R. Manfredi J. Prives C. (2016). The tail that wags the dog: how the disordered C-terminal domain controls the transcriptional activities of the p53 tumor-suppressor protein. Trends Biochem. Sci. 41 (12), 1022–1034. 10.1016/j.tibs.2016.08.011 27669647 PMC6258061

[B102] Leitner A. Faini M. Stengel F. Aebersold R. (2016). Crosslinking and mass spectrometry: an integrated technology to understand the structure and function of molecular machines. Trends Biochem. Sci. 41 (1), 20–32. 10.1016/j.tibs.2015.10.008 26654279

[B103] Leitner A. Joachimiak L. A. Unverdorben P. Walzthoeni T. Frydman J. Förster F. (2014). Chemical cross-linking/mass spectrometry targeting acidic residues in proteins and protein complexes. Proc. Natl. Acad. Sci. USA 111 (26), 9455–9460. 10.1073/pnas.1320298111 24938783 PMC4084482

[B104] Leitner A. Reischl R. Walzthoeni T. Herzog F. Bohn S. Förster F. (2012). Expanding the chemical cross-linking toolbox by the use of multiple proteases and enrichment by size exclusion chromatography. Mol. Cell Proteomics 11 (3), 014126. 10.1074/mcp.M111.014126 22286754 PMC3316732

[B105] Leney A. C. Heck A. J. (2017). Native mass spectrometry: what is in the name?. J. Am. Soc. Mass Spectrom. 28 (1), 5–13. 10.1007/s13361-016-1545-3 PMC5174146 27909974

[B106] Leuenberger P. Ganscha S. Kahraman A. Cappelletti V. Boersema P. J. von Mering C. (2017). Cell-wide analysis of protein thermal unfolding reveals determinants of thermostability. Science 355 (6327), eaai7825. 10.1126/science.aai7825 28232526

[B107] Li A. Christensen H. M. Stewart L. R. Roth K. A. Chiesa R. Harris D. A. (2007). Neonatal lethality in transgenic mice expressing prion protein with a deletion of residues 105–125. EMBO J. 26 (2), 548–558. 10.1038/sj.emboj.7601507 17245437 PMC1783448

[B108] Li K. S. Rempel D. L. Gross M. L. (2016). Conformational-sensitive fast photochemical oxidation of proteins and mass spectrometry characterize amyloid beta 1–42 aggregation. J. Am. Chem. Soc. 138 (37), 12090–12098. 10.1021/jacs.6b07543 27568528 PMC5221481

[B109] Liu F. Lössl P. Scheltema R. Viner R. Heck A. J. R. (2017). Optimized fragmentation schemes and data analysis strategies for proteome-wide cross-link identification. Nat. Commun. 8, 15473. 10.1038/ncomms15473 28524877 PMC5454533

[B110] Liu X. R. Zhang M. M. Gross M. L. (2020). Mass spectrometry-based protein footprinting for higher-order structure analysis: fundamentals and applications. Chem. Rev. 120 (10), 4355–4454. 10.1021/acs.chemrev.9b00815 32319757 PMC7531764

[B111] Liu Z. Huang Y. (2014). Advantages of proteins being disordered. Protein Sci. 23 (5), 539–550. 10.1002/pro.2443 24532081 PMC4005706

[B112] Liuni P. Rob T. Wilson D. J. (2010). A microfluidic reactor for rapid, low-pressure proteolysis with on-chip electrospray ionization. Rapid Commun. Mass Spectrom. 24 (3), 315–320. 10.1002/rcm.4391 20049884

[B113] Mädler S. Bich C. Touboul D. Zenobi R. (2009). Chemical cross-linking with NHS esters: a systematic study on amino acid reactivities. J. Mass Spectrom. 44 (5), 694–706. 10.1002/jms.1544 19132714

[B114] Martin E. M. Jackson M. P. Gamerdinger M. Gense K. Karamonos T. K. Humes J. R. (2018). Conformational flexibility within the nascent polypeptide-associated complex enables its interactions with structurally diverse client proteins. J. Biol. Chem. 293 (22), 8554–8568. 10.1074/jbc.RA117.001568 29650757 PMC5986199

[B115] McDonald A. J. Leon D. R. Markham K. A. Wu B. Heckendorf C. F. Schilling K. (2019). Altered domain structure of the prion protein caused by Cu2+ binding and functionally relevant mutations: analysis by cross-linking, MS/MS, and NMR. Structure 27 (6), 907–e5. 10.1016/j.str.2019.03.008 30956132 PMC6736647

[B116] Mead S. (2006). Prion disease genetics. Eur. J. Hum. Genet. 14 (3), 273–281. 10.1038/sj.ejhg.5201544 16391566

[B117] Meinen B. A. Gadkari V. V. Stull F. Ruotolo B. T. Bardwell J. C. A. (2019). SERF engages in a fuzzy complex that accelerates primary nucleation of amyloid proteins. Proc. Natl. Acad. Sci. USA 116 (46), 23040–23049. 10.1073/pnas.1913316116 31659041 PMC6859325

[B118] Mendes M. L. Fischer L. Chen Z. A. Barbon M. O'Reilly F. J. Giese S. H. (2019). An integrated workflow for crosslinking mass spectrometry. Mol. Syst. Biol. 15 (9), e8994. 10.15252/msb.20198994 31556486 PMC6753376

[B119] Minde D. P. Ramakrishna M. Lilley K. S. (2020). Biotin proximity tagging favours unfolded proteins and enables the study of intrinsically disordered regions. Commun. Biol. 3 (1), 38. 10.1038/s42003-020-0758-y 31969649 PMC6976632

[B120] Mirbaha H. Chen D. Morazova O. A. Ruff K. M. Sharma A. M. Liu X. (2018). Inert and seed-competent tau monomers suggest structural origins of aggregation. eLife 7, e36584. 10.7554/eLife.36584 29988016 PMC6039173

[B121] Mittal A. Lyle N. Harmon T. S. Pappu R. V. (2014). Hamiltonian switch metropolis Monte Carlo simulations for improved conformational sampling of intrinsically disordered regions tethered to ordered domains of proteins. J. Chem. Theor. Comput 10 (8), 3550–3562. 10.1021/ct5002297 PMC4132852 25136274

[B122] Murata K. Wolf M. (2018). Cryo-electron microscopy for structural analysis of dynamic biological macromolecules. Biochim. Biophys. Acta (BBA) - Gen. Subjects 1862 (2), 324–334. 10.1016/j.bbagen.2017.07.020 28756276

[B123] Murphy M. P. LeVine H. III (2010). Alzheimer’s disease and the amyloid-beta peptide. J. Alzheimers Dis. 19 (1), 311–323. 10.3233/JAD-2010-1221 20061647 PMC2813509

[B124] Mylonas E. Hascher A. Bernadó P. Blackledge M. Mandelkow E. Svergun D. I. (2008). Domain conformation of tau protein studied by solution small-angle X-ray scattering. Biochemistry 47 (39), 10345–10353. 10.1021/bi800900d 18771286

[B125] Narayanan R. L. Dürr U. H. Bibow S. Biernat J. Mandelkow E. Zweckstetter M. (2010). Automatic assignment of the intrinsically disordered protein tau with 441-residues. J. Am. Chem. Soc. 132 (34), 11906–11907. 10.1021/ja105657f 20687558

[B126] Natalello A. Santambrogio C. Grandori R. (2017). Are charge-state distributions a reliable tool describing molecular ensembles of intrinsically disordered proteins by native MS?. J. Am. Soc. Mass Spectrom. 28 (1), 21–28. 10.1007/s13361-016-1490-1 27730522

[B127] Nath A. Sammalkorpi M. DeWitt D. C. Trexler A. J. Elbaum-Garfinkle S. O’Hern C. S. (2012). The conformational ensembles of α-synuclein and tau: combining single-molecule FRET and simulations. Biophys. J. 103 (9), 1940–1949. 10.1016/j.bpj.2012.09.032 23199922 PMC3491709

[B128] Niu B. Mackness B. C. Rempel D. L. Zhang H. Cui W. Matthews C. R. (2017). Incorporation of a reporter peptide in FPOP Compensates for adventitious scavengers and permits time-dependent measurements. J. Am. Soc. Mass Spectrom. 28 (2), 389–392. 10.1007/s13361-016-1552-4 27924496 PMC5233597

[B129] Nshanian M. Lantz C. Wongkongkathep P. Schrader T. Klärner F. G. Blümke A. (2019). Native top-down mass spectrometry and ion mobility spectrometry of the interaction of tau protein with a molecular tweezer assembly modulator. J. Am. Soc. Mass Spectrom. 30 (1), 16–23. 10.1007/s13361-018-2027-6 30062477 PMC6320309

[B130] Oldfield C. J. Cheng Y. Cortese M. S. Brown C. J. Uversky V. N. Dunker A. K. (2005). Comparing and combining predictors of mostly disordered proteins. Biochemistry 44 (6), 1989–2000. 10.1021/bi047993o 15697224

[B131] Orbán-Németh Z. Beveridge R. Hollenstein D. M. Rampler E. Stranzl T. Hudecz O. (2018). Structural prediction of protein models using distance restraints derived from cross-linking mass spectrometry data. Nat. Protoc. 13 (3), 478–494. 10.1038/nprot.2017.146 29419816 PMC5999019

[B132] O’Reilly F. J. Xue L. Graziadei A. Sinn L. Lenz S. Tegunov D. (2020). In-cell architecture of an actively transcribing-translating expressome. Science 369 (6503), 554–557. 32732422 10.1126/science.abb3758 PMC7115962

[B133] Parker B. W. Goncz E. J. Krist D. T. Statsyuk A. V. Nesvizhskii A. I. Weiss E. L. (2019). Mapping low-affinity/high-specificity peptide-protein interactions using ligand-footprinting mass spectrometry. Proc. Natl. Acad. Sci. USA 116 (42), 21001–21011. 10.1073/pnas.1819533116 31578253 PMC6800362

[B134] Peng Y. Cao S. Kiselar J. Xiao X. Du Z. Hsieh A. (2019). A metastable contact and structural disorder in the estrogen receptor transactivation domain. Structure 27 (2), 229–e224. 10.1016/j.str.2018.10.026 30581045 PMC6365180

[B135] Peng Z. Yan J. Fan X. Mizianty M. J. Xue B. Wang K. (2015). Exceptionally abundant exceptions: comprehensive characterization of intrinsic disorder in all domains of life. Cell Mol Life Sci 72 (1), 137–151. 10.1007/s00018-014-1661-9 24939692 PMC11113594

[B136] Politis A. Schmidt C. (2018). Structural characterisation of medically relevant protein assemblies by integrating mass spectrometry with computational modelling. J. Proteomics 175, 34–41. 10.1016/j.jprot.2017.04.019 28461040

[B137] Popov K. I. Makepeace K. A. T. Petrotchenko E. V. Dokholyan N. V. Borchers C. H. (2019). Insight into the structure of the "unstructured" tau protein. Structure 27 (11), 1710–e1714. 10.1016/j.str.2019.09.003 31628033

[B138] Preston G. W. Radford S. E. Ashcroft A. E. Wilson A. J. (2014). Analysis of amyloid nanostructures using photo-cross-linking: *in situ* comparison of three widely used photo-cross-linkers. ACS Chem. Biol. 9 (3), 761–768. 10.1021/cb400731s 24372480 PMC3964826

[B139] Ramsden M. Kotilinek L. Forster C. Paulson J. McGowan E. SantaCruz K. (2005). Age-Dependent neurofibrillary tangle formation, neuron loss, and memory impairment in a mouse model of human tauopathy (P301L). J. Neurosci. 25 (46), 10637–10647. 10.1523/JNEUROSCI.3279-05.2005 16291936 PMC6725849

[B140] Rappsilber J. (2011). The beginning of a beautiful friendship: cross-linking/mass spectrometry and modelling of proteins and multi-protein complexes. J. Struct. Biol. 173 (3), 530–540. 10.1016/j.jsb.2010.10.014 21029779 PMC3043253

[B141] Ridgeway M. E. Lubeck M. Jordens J. Mann M. Park M. A. (2018). Trapped ion mobility spectrometry: a short review. Int. J. Mass Spectrom. 425, 22–35. 10.1016/j.ijms.2018.01.006

[B142] Riek R. Hornemann S. Wider G. Billeter M. Glockshuber R. Wüthrich K. (1996). NMR structure of the mouse prion protein domain PrP(121–231). Nature 382 (6587), 180–182. 10.1038/382180a0 8700211

[B143] Rist W. Rodriguez F. Jørgensen T. J. Mayer M. P. (2005). Analysis of subsecond protein dynamics by amide hydrogen exchange and mass spectrometry using a quenched-flow setup. Protein Sci. 14 (3), 626–632. 10.1110/ps.041098305 15689511 PMC2279298

[B144] Rizzu P. Van Swieten J. C. Joosse M. Hasegawa M. Stevens M. Tibben A. (1999). High prevalence of mutations in the microtubule-associated protein tau in a population study of frontotemporal dementia in Netherlands. Am. J. Hum. Genet. 64 (2), 414–421. 10.1086/302256 9973279 PMC1377751

[B145] Rob T. Liuni P. Gill P. K. Zhu S. Balachandran N. Berti P. J. (2012). Measuring dynamics in weakly structured regions of proteins using microfluidics-enabled subsecond H/D exchange mass spectrometry. Anal. Chem. 84 (8), 3771–3779. 10.1021/ac300365u 22458633

[B146] Rout M. P. Sali A. (2019). Principles for integrative structural biology studies. Cell 177 (6), 1384–1403. 10.1016/j.cell.2019.05.016 31150619 PMC6810593

[B147] Ruotolo B. T. Benesch J. L. Sandercock A. M. Hyung S. J. Robinson C. V. (2008). Ion mobility-mass spectrometry analysis of large protein complexes. Nat. Protoc. 3 (7), 1139–1152. 10.1038/nprot.2008.78 18600219

[B148] Rusinga F. I. Weis D. D. (2017a). Automated strong Cation-exchange Cleanup to remove macromolecular crowding agents for protein hydrogen exchange mass spectrometry. Anal. Chem. 89 (2), 1275–1282. 10.1021/acs.analchem.6b04057 27936623

[B149] Rusinga F. I. Weis D. D. (2017b). Soft interactions and volume exclusion by polymeric crowders can stabilize or destabilize transient structure in disordered proteins depending on polymer concentration. Proteins 85 (8), 1468–1479. 10.1002/prot.25307 28425679

[B150] Saikusa K. Nagadoi A. Hara K. Fuchigami S. Kurumizaka H. Nishimura Y. (2015). Mass spectrometric approach for Characterizing the disordered tail regions of the histone H2A/H2B dimer. Anal. Chem. 87 (4), 2220–2227. 10.1021/ac503689w 25594579

[B151] Santambrogio C. Natalello A. Brocca S. Ponzini E. Grandori R. (2019). Conformational characterization and classification of intrinsically disordered proteins by native mass spectrometry and charge‐state distribution analysis. Proteomics 19 (6), 1800060. 10.1002/pmic.201800060 30365227

[B152] Schmidt C. Macpherson J. A. Lau A. M. Tan K. W. Fraternali F. Politis A. (2017). Surface accessibility and dynamics of macromolecular assemblies probed by covalent labeling mass spectrometry and integrative modeling. Anal. Chem. 89 (3), 1459–1468. 10.1021/acs.analchem.6b02875 28208298 PMC5299547

[B153] Schmidt C. Robinson C. V. (2014). A comparative cross-linking strategy to probe conformational changes in protein complexes. Nat. Protoc. 9 (9), 2224–2236. 10.1038/nprot.2014.144 25144272 PMC4172966

[B154] Schmidt C. Zhou M. Marriott H. Morgner N. Politis A. Robinson C. V. (2013). Comparative cross-linking and mass spectrometry of an intact F-type ATPase suggest a role for phosphorylation. Nat. Commun. 4, 1985. 10.1038/ncomms2985 23756419 PMC3709506

[B155] Schmidt R. Sinz A. (2017). Improved single-step enrichment methods of cross-linked products for protein structure analysis and protein interaction mapping. Anal. Bioanal. Chem. 409 (9), 2393–2400. 10.1007/s00216-017-0185-1 28083664

[B156] Schneider M. Belsom A. Rappsilber J. (2018). Protein tertiary structure by crosslinking/mass spectrometry. Trends Biochem. Sci. 43 (3), 157–169. 10.1016/j.tibs.2017.12.006 29395654 PMC5854373

[B157] Schwalbe M. Ozenne V. Bibow S. Jaremko M. Jaremko L. Gajda M. (2014). Predictive atomic resolution descriptions of intrinsically disordered hTau40 and α-synuclein in solution from NMR and small angle scattering. Structure 22 (2), 238–249. 10.1016/j.str.2013.10.020 24361273

[B158] Semmler L. Reiter-Brennan C. Klein A. (2019). BRCA1 and breast cancer: a review of the underlying mechanisms resulting in the tissue-specific tumorigenesis in mutation Carriers. J. Breast Cancer 22 (1), 1–14. 10.4048/jbc.2019.22.e6 30941229 PMC6438831

[B159] Sharma R. Raduly Z. Miskei M. Fuxreiter M. (2015). Fuzzy complexes: specific binding without complete folding. FEBS Lett. 589 (19), 2533–2542. 10.1016/j.febslet.2015.07.022 26226339

[B160] Sharon M. Robinson C. V. (2007). The role of mass spectrometry in structure elucidation of dynamic protein complexes. Annu. Rev. Biochem. 76 (1), 167–193. 10.1146/annurev.biochem.76.061005.090816 17328674

[B161] Sinha S. Lopes D. H. Du Z. Pang E. S. Shanmugam A. Lomakin A. (2011). Lysine-specific molecular tweezers are broad-spectrum inhibitors of assembly and toxicity of amyloid proteins. J. Am. Chem. Soc. 133 (42), 16958–16969. 10.1021/ja206279b 21916458 PMC3210512

[B162] Sinz A. Arlt C. Chorev D. Sharon M. (2015). Chemical cross-linking and native mass spectrometry: a fruitful combination for structural biology. Protein Sci. 24 (8), 1193–1209. 10.1002/pro.2696 25970732 PMC4534171

[B163] Sinz A. (2014). The advancement of chemical cross-linking and mass spectrometry for structural proteomics: from single proteins to protein interaction networks. Expert Rev. Proteomics 11 (6), 733–743. 10.1586/14789450.2014.960852 25227871

[B164] Skinner O. S. Haverland N. A. Fornelli L. Melani R. D. Do Vale L. H. F. Seckler H. S. (2017). Top-down characterization of endogenous protein complexes with native proteomics. Nat. Chem. Biol. 14, 36. 10.1038/nchembio.2515 29131144 PMC5726920

[B165] Snijder J. van de Waterbeemd M. Glover M. S. Shi L. Clemmer D. E. Heck A. J. (2015). Conformational landscape and pathway of disulfide bond reduction of human alpha defensin. Protein Sci. 24 (8), 1264–1271. 10.1002/pro.2694 25970658 PMC4534177

[B166] Spevacek A. R. Evans E. G. Miller J. L. Meyer H. C. Pelton J. G. GlennMillhauser L. M. G. L. (2013). Zinc drives a tertiary fold in the prion protein with familial disease mutation sites at the interface. Structure 21 (2), 236–246. 10.1016/j.str.2012.12.002 23290724 PMC3570608

[B167] Steigenberger B. Pieters R. J. Heck A. J. R. Scheltema R. A. (2019). PhoX: an IMAC-enrichable cross-linking reagent. ACS Cent. Sci. 5 (9), 1514–1522. 10.1021/acscentsci.9b00416 31572778 PMC6764163

[B168] Stephani M. Picchianti L. Gajic A. Beveridge R. Skarwan E. Sanchez de Medina Hernandez V. (2020). A cross-kingdom conserved ER-phagy receptor maintains endoplasmic reticulum homeostasis during stress. Elife 9. 10.7554/eLife.58396 PMC7515635 32851973

[B169] Stephens A. D. Zacharopoulou M. Moons R. Fusco G. Chiki N. (2020). Extent of N-terminus exposure of monomeric alpha-synuclein determines its aggregation propensity. Nat. Commun. 11 (1), 2820. 10.1038/s41467-020-16564-3 32499486 PMC7272411

[B170] Stuchfield D. Barran P. (2018). Unique insights to intrinsically disordered proteins provided by ion mobility mass spectrometry. Curr. Opin. Chem. Biol. 42, 177–185. 10.1016/j.cbpa.2018.01.007 29428839

[B171] Suryadinata R. Sadowski M. Sarcevic B. (2010). Control of cell cycle progression by phosphorylation of cyclin-dependent kinase (CDK) substrates. Biosci. Rep. 30 (4), 243–255. 10.1042/BSR20090171 20337599

[B172] Svejdal R. R. Dickinson E. R. Sticker D. Kutter J. P. Rand K. D. (2019). Thiol-ene microfluidic chip for performing hydrogen/deuterium exchange of proteins at subsecond time scales. Anal. Chem. 91 (2), 1309–1317. 10.1021/acs.analchem.8b03050 30525463

[B173] Takamori S. Holt M. Stenius K. Lemke E. A. Grønborg M. Riedel D. (2006). Molecular anatomy of a trafficking organelle. Cell 127 (4), 831–846. 10.1016/j.cell.2006.10.030 17110340

[B174] Takamoto K. Chance M. R. (2006). Radiolytic protein footprinting with mass spectrometry to probe the structure of macromolecular complexes. Annu. Rev. Biophys. Biomol. Struct. 35, 251–276. 10.1146/annurev.biophys.35.040405.102050 16689636

[B175] Testa L. Brocca S. Grandori R. (2011). Charge-surface correlation in electrospray ionization of folded and unfolded proteins. Anal. Chem. 83 (17), 6459–6463. 10.1021/ac201740z 21800882

[B176] Thielges M. C. Zimmermann J. Yu W. Oda M. Romesberg F. E. (2008). Exploring the energy landscape of antibody-antigen complexes: protein dynamics, flexibility, and molecular recognition. Biochemistry 47 (27), 7237–7247. 10.1021/bi800374q 18549243

[B177] Tidow H. Melero R. Mylonas E. Freund S. M. Grossmann J. G. Carazo J. M. (2007). Quaternary structures of tumor suppressor p53 and a specific p53 DNA complex. Proc. Natl. Acad. Sci. USA 104 (30), 12324–12329. 10.1073/pnas.0705069104 17620598 PMC1941468

[B178] Trabjerg E. Nazari Z. E. Rand K. D. (2018). Conformational analysis of complex protein states by hydrogen/deuterium exchange mass spectrometry (HDX-MS): challenges and emerging solutions. Trac Trends Anal. Chem. 106, 125–138. 10.1016/j.trac.2018.06.008

[B179] Ujma J. Ropartz D. Giles K. Richardson K. Langridge D. Wildgoose J. (2019). Cyclic ion mobility mass spectrometry distinguishes anomers and open-ring forms of pentasaccharides. J. Am. Soc. Mass Spectrom. 30 (6), 1028–1037. 10.1007/s13361-019-02168-9 30977045 PMC6517361

[B180] Uversky V. N. Oldfield C. J. Dunker A. K. (2008). Intrinsically disordered proteins in human diseases: introducing the D2 Concept. Annu. Rev. Biophys. 37 (1), 215–246. 10.1146/annurev.biophys.37.032807.125924 18573080

[B181] Vahidi S. Konermann L. (2016). Probing the time scale of FPOP (fast photochemical oxidation of proteins): radical reactions extend over tens of milliseconds. J. Am. Soc. Mass Spectrom. 27 (7), 1156–1164. 10.1007/s13361-016-1389-x 27067899

[B182] Van der Rest G. Rezaei H. Halgand F. (2017). Monitoring conformational landscape of ovine prion protein monomer using ion mobility coupled to mass spectrometry. J. Am. Soc. Mass Spectrom. 28 (2), 303–314. 10.1007/s13361-016-1522-x 27757822

[B183] Vassilev L. T. Vu B. T. Graves B. Carvajal D. Podlaski F. Filipovic Z. (2004). *In Vivo* activation of the p53 pathway by small-molecule antagonists of MDM2. Science 303 (5659), 844–848. 10.1126/science.1092472 14704432

[B184] Vimer S. Ben-Nissan G. Sharon M. (2020). Direct characterization of overproduced proteins by native mass spectrometry. Nat. Protoc. 15 (2), 236–265. 10.1038/s41596-019-0233-8 31942081 PMC7212079

[B185] Vöpel T. Bravo-Rodriguez K. Mittal S. Vachharajani S. Gnutt D. Sharma A. (2017). Inhibition of huntingtin exon-1 aggregation by the molecular tweezer CLR01. J. Am. Chem. Soc. 139 (16), 5640–5643. 10.1021/jacs.6b11039 28406616 PMC5506490

[B186] Walters B. T. Mayne L. Hinshaw J. R. Sosnick T. R. Englander S. W. (2013). Folding of a large protein at high structural resolution. Proc. Natl. Acad. Sci. USA 110 (47), 18898–18903. 10.1073/pnas.1319482110 24191053 PMC3839771

[B187] Wang L. Chance M. R. (2017). Protein footprinting comes of age: mass spectrometry for biophysical structure assessment. Mol. Cell Proteomics 16 (5), 706–716. 10.1074/mcp.O116.064386 28275051 PMC5417815

[B188] Wang L. Chance M. R. (2011). Structural mass spectrometry of proteins using hydroxyl radical based protein footprinting. Anal. Chem. 83 (19), 7234–7241. 10.1021/ac200567u 21770468 PMC3184339

[B189] Webb B. Viswanath S. Bonomi M. Pellarin R. Greenberg C. H. Saltzberg D. (2018). Integrative structure modeling with the integrative modeling platform. Protein Sci. 27 (1), 245–258. 10.1002/pro.3311 28960548 PMC5734277

[B190] Wittig S. Haupt C. Hoffmann W. Kostmann S. Pagel K. Schmidt C. (2019). Oligomerisation of synaptobrevin-2 studied by native mass spectrometry and chemical cross-linking. J. Am. Soc. Mass Spectrom. 30 (1), 149–160. 10.1007/s13361-018-2000-4 29949059 PMC6318248

[B191] Wongkongkathep P. Han J. Y. Choi T. S. Yin S. Kim H. I. Loo J. A. (2018). Native top-down mass spectrometry and ion mobility MS for Characterizing the cobalt and manganese metal binding of α-synuclein protein. J. Am. Soc. Mass Spectrom. 29 (9), 1870–1880. 10.1007/s13361-018-2002-2 29951842 PMC6087494

[B192] Wright P. E. Dyson H. J. (2015). Intrinsically disordered proteins in cellular signalling and regulation. Nat. Rev. Mol. Cell Biol 16 (1), 18–29. 10.1038/nrm3920 25531225 PMC4405151

[B193] Wright P. E. Dyson H. J. (2009). Linking folding and binding. Curr. Opin. Struct. Biol. 19 (1), 31–38. 10.1016/j.sbi.2008.12.003 19157855 PMC2675572

[B194] Wu B. McDonald A. J. Markham K. Rich C. B. McHugh K. P. Tatzelt J. (2017). The N-terminus of the prion protein is a toxic effector regulated by the C-terminus. eLife 6, e23473. 10.7554/eLife.23473 28527237 PMC5469617

[B195] Xie Y. Zhang J. Yin S. Loo J. A. (2006). Top-down ESI-ECD-FT-ICR mass spectrometry localizes noncovalent protein-ligand binding sites. J. Am. Chem. Soc. 128 (45), 14432–14433. 10.1021/ja063197p 17090006

[B196] Xu G. Chance M. R. (2007). Hydroxyl radical-mediated modification of proteins as probes for structural proteomics. Chem. Rev. 107 (8), 3514–3543. 10.1021/cr0682047 17683160

[B197] Yamamoto T. Shimizu Y. Ueda T. Shiro Y. Suematsu M. (2011). Application of micro-reactor chip technique for millisecond quenching of deuterium incorporation into 70S ribosomal protein complex. Int. J. Mass Spectrom. 302 (1), 132–138. 10.1016/j.ijms.2010.08.029

[B198] Yan Y. Chen G. Wei H. Huang R. Y. Mo J. Rempel D. L. (2014). Fast photochemical oxidation of proteins (FPOP) maps the epitope of EGFR binding to adnectin. J. Am. Soc. Mass Spectrom. 25 (12), 2084–2092. 10.1007/s13361-014-0993-x 25267085 PMC4224620

[B199] Yu C. Huang L. (2018). Cross-Linking mass spectrometry: an emerging technology for interactomics and structural biology. Anal. Chem. 90, 144–165. 10.1021/acs.analchem.7b04431 29160693 PMC6022837

[B200] Zahn R. Liu A. Lührs T. Riek R. von Schroetter C. López García F. (2000). NMR solution structure of the human prion protein. Proc. Natl. Acad. Sci. USA 97 (1), 145–150. 10.1073/pnas.97.1.145 10618385 PMC26630

[B201] Zhang Y. Rempel D. L. Zhang J. Sharma A. K. Mirica L. M. Gross M. L. (2013). Pulsed hydrogen-deuterium exchange mass spectrometry probes conformational changes in amyloid beta (Aβ) peptide aggregation. Proc. Natl. Acad. Sci. USA 110 (36), 14604–14609. 10.1073/pnas.1309175110 23959898 PMC3767558

[B202] Zheng X. Liu D. Klärner F. G. Schrader T. Bitan G. Bowers M. T. (2015). Amyloid β-protein assembly: the effect of molecular tweezers CLR01 and CLR03. J. Phys. Chem. B 119 (14), 4831–4841. 10.1021/acs.jpcb.5b00692 25751170 PMC4415044

[B203] Zheng X. Wu C. Liu D. Li H. Bitan G. Shea J. E. (2016). Mechanism of C-terminal fragments of amyloid β-protein as Aβ inhibitors: do C-terminal interactions play a key role in their inhibitory activity?. J. Phys. Chem. B 120 (8), 1615–1623. 10.1021/acs.jpcb.5b08177 26439281 PMC4777659

[B204] Ziemianowicz D. S. MacCallum J. L. Schriemer D. C. (2020). Correlation between labeling yield and surface accessibility in covalent labeling mass spectrometry. J. Am. Soc. Mass Spectrom. 31 (2), 207–216. 10.1021/jasms.9b00083 32031402

